# Ionized Keplerian Disks Demonstrating Interplay Between Strong Gravity and Magnetism

**DOI:** 10.3390/e27121253

**Published:** 2025-12-12

**Authors:** Zdeněk Stuchlík, Jaroslav Vrba

**Affiliations:** Research Centre for Theoretical Physics and Astrophysics, Institute of Physics, Silesian University in Opava, Bezručovo nám. 13, 746 01 Opava, Czech Republic; zdenek.stuchlik@physics.slu.cz

**Keywords:** extremely compact objects, dipole magnetic field, strong gravity and magnetism, charged particles, Keplerian disks, ionization

## Abstract

Using the dynamics of charged test particles, we study the interplay of extremely strong gravitational and magnetic fields acting on ionized Keplerian disks. We assume a Schwarzschild spacetime of mass *M* combined with a dipole magnetic field represented by a dimensionless parameter *b*, characterizing the influence of fields near the gravitational radius $r_{g}=2GM/c^{2}$. The particle dynamics can be realized in three regimes: gravitational (b≪1), magnetic (b≫1), and chaotic (b∼1). We demonstrate the ionization of disks that are originally both orthogonal and inclined to the magnetic field axis and consider both magnetic attraction and magnetic repulsion acting on the ionized particles. The case of secondary ionized equatorial charged disks is also discussed. The ionization in the dipole magnetic field is compared with the case of a Schwarzschild spacetime endowed with an asymptotically uniform magnetic field. The differences in the dipole and uniform fields are significant in the magnetic and chaotic regimes, while they are suppressed in the gravitational regime.

## 1. Introduction

The interplay between the strong gravitational and electromagnetic fields that are observed around neutron stars or black holes (or, alternatively, their mimickers) in binary systems [1], or around supermassive black holes in active galactic nuclei [2], can be represented effectively through the behavior of ionized Keplerian disks. This approach is based on the modification of standard, geometrically thin Keplerian disks [3], formed by effectively (globally) uncharged matter, via the effects of external, large-scale magnetic fields after a plausible astrophysical ionization process acting on the disk matter.

We can consider several ionization processes [4]. The most natural one is the ionization caused by the irradiation of atoms in the Keplerian disk, where electrons of the atomic structure escape due to the capture of photons with sufficiently high energies. Such captured photons can start the ionization process or increase its extent. The sources of these photons can be found in the disk corona, as in models of the generation of profiled spectral lines [5,6,7]. Another means of ionization is the decay of neutrons contained in the accretion disk [8]. Finally, in the innermost parts of Keplerian disks, near their inner edges, where matter starts to fall freely onto the central compact object, a decrease in plasma density can trigger the effective action of the electromagnetic forces of the external magnetic field, which separates protons (ions) from electrons, demonstrating the ionization of the disk.

The ionization of Keplerian disks has been considered regarding Schwarzschild or Kerr black holes immersed in an asymptotically uniform magnetic field [4,9] or regarding an external parabolic magnetic field [10]. The relative importance of the gravitational and electromagnetic forces in the vicinity of the compact object can be characterized by the so-called magnetic parameter *b*, reflecting the relative magnitudes of these forces [9], by comparing the Keplerian frequency related to its gravitational field in the vicinity of the compact object,

(1) $$\Omega_{K}=\frac{r_{g}^{1/2}c}{r^{3/2}},$$

where $r_{g}=MG/c^{2}$ is the gravitational radius of the compact object, and the cyclotron frequency related to the Lorentz force acting in the considered magnetic field near the compact object is

(2) $$\Omega_{c}=\frac{qBc}{E},$$

where $E=\gamma mc^{2}$ is the particle energy. In relation to the Keplerian frequency, which is considered to be positive, the cyclotron frequency can be positive or negative depending on the orientation of the magnetic field and the sign of the particle’s electric charge. In the vicinity of the gravitational radius, $r∼r_{g}$, we arrive at $\Omega_{K}∼c^{3}/\left(GM\right)$. The assumption of the Lorentz factor γ∼1 leads to the expression $\Omega_{c}=\frac{qB}{mc}$. The relation among the magnitudes of these frequencies determines the dimensionless magnetic parameter *b*, serving as a constant for the motion of charged test particles. For a black hole immersed in an asymptotically uniform magnetic field, we thus arrive at

(3) $$b_{u}=\frac{qBMG}{mc^{4}}$$

which characterizes the relative strength of the Lorentz and gravitational forces acting on the particles in close proximity to the black hole [11,12]. A similar constant of motion can be introduced for motion in different magnetic fields and other compact objects.

The three regimes of motion in the vicinity of the black hole immersed in a uniform magnetic field can be expressed in terms of the relation among the Keplerian and cyclotron (Larmor) frequencies (or the ratio of the gravitational and electromagnetic forces): a gravitational regime where $\Omega_{K}\gg \Omega_{c}$ ($|b_{u}|\ll 1$); a magnetic regime where $\Omega_{K}\ll \Omega_{c}$ ($|b_{u}|\gg 1$); and a chaotic regime where $\Omega_{K}∼\Omega_{c}$ ($|b_{u}|∼1$). The same relations hold in the case of other magnetic fields with a suitably defined magnetic parameter *b*, considered in the vicinity of the compact object. It must be noted that, with increasing distances from the central compact object, the regime of particle motion is modified in accordance with the radial dependence of the gravitational and electromagnetic forces [4,13].

Here, we focus on motion in a dipole magnetic field in the Schwarzschild spacetime, using our previous results given in [13]. This simple (but fully general-relativistic) model is highly suitable to illustrate the interplay of the gravitational and electromagnetic forces in the vicinities of compact objects through the fates of ionized Keplerian disks. This is because the motion of charged particles, which occurs due to the ionization of the disks, can be treated with simple semi-analytic methods. The fate of the disk will be represented in terms of the magnetic parameter *b* directly governing the interplay of the forces in close proximity to the compact object. As the boundary of the compact object, the gravitational radius of the Schwarzschild geometry (or its immediate vicinity) can be considered.

Magnetized compact objects represented by the Schwarzschild geometry describing the gravity of mass parameter *M*, combined with the dipole magnetic field with dipole magnetic moment μ, were introduced in [14,15] for the case of a current loop orbiting a Schwarzschild black hole in an accretion disk. However, these can also be considered in more general situations, e.g., in the case of magnetized neutron stars [16,17,18]. Of special interest are black hole mimickers, e.g., gravastars [19,20,21,22,23].

Regarding neutron stars, extremely strong magnetic fields are observed near their surfaces—the magnetic field intensity ranges from $10^{8}$ G in the old binary systems to $10^{15}$ G in young magnetars [24]. Slightly weaker magnetic fields are observed around black holes, where the magnetic field intensity reaches up to $10^{8}$ G in microquasars or can reach up to $10^{5}$ G around supermassive BHs in active galactic nuclei [25,26,27,28]. Much weaker magnetic fields can be expected around isolated black holes or around hypothetical black hole mimickers.

A detailed study of the charged particle motion against such a background was realized for neutron stars under the assumption of surfaces located at the radius R=3M, giving the upper limit of the so-called extremely compact objects with an external Schwarzschild geometry [13]. At R=3M, the photon sphere of vacuum Schwarzschild spacetimes can be found; unusual behavior regarding the forces acting on particles moving in the region of 2M<r<3M was shown in [29]. Studies related to neutron stars are extended here to the case of black hole mimickers with R∼2M. The results of these studies of charged particle motion that appear to be most relevant to our investigations are related to the possible existence of “in”- and “off”-equatorial circular orbits and the chaotic motion in belts that is concentrated around circular orbits. For corotating charged particles, both “in” and “off” circular orbits can exist only for particles under repulsive Lorentz forces, while only “in” orbits are possible under attractive Lorentz forces. (The opposite holds for counter-rotating particles, as related to the static distant observers). Around stable circular orbits, epicyclic oscillatory motion of a harmonic (or nearly harmonic) nature occurs. This type of motion can be realized in all three regimes if concentrated in sufficiently close proximity to the circular orbit. “Off”-equatorial circular orbits play an important role in our study of ionized Keplerian disks in regard to the repulsive magnetic influence. For a given, sufficiently low magnetic parameter *b*, they exist within the equatorial circular orbit and are marginally stable against vertical perturbations, and they can approach the radius r∼2M along the symmetry axis of the magnetic field; above (under) the radius r=3M, these “off”-equatorial orbits are stable (unstable) against perturbations. Chaotic particle motion occurs in belts, being concentrated around the “off”-equatorial circular orbits [13], resulting in strong imprints in the behavior of ionized Keplerian disks.

In the present paper, we generalize the results of [13], extending the study to the region located under the photon circular orbit. However, it must be noted that the focus of the present work is on collective phenomena related to charged particle motion, realized by considering ionized Keplerian disks that could be of direct astrophysical relevance. Our analysis of ionized Keplerian disks is presented in a general manner and can be appropriately applied in systems containing black holes or their mimickers at the region of r>2M. On the other hand, for binary systems containing neutron stars, the applicability of our work is restricted to the region of r>3M due to the radius of the observed neutron stars, which exceeds the photon sphere. In modeling ionized Keplerian disks, we abandon the influence of the radiation of the charged particles on their motion, using the Lorentz equations of motion only. The inclusion of the Lorentz–Dirac equation requires extensive numerical calculations and the exact specification of the astrophysical conditions—such work is planned for the future.

Throughout this paper, we use a space-like signature (−,+,+,+) and a geometric system of units in which G=c=1. Greek indices span 0–3 and Latin indices span 1–3.

## 2. Compact Objects with Dipole Magnetosphere

The simplest fully relativistic approximation of magnetized extremely compact objects is given by the Schwarzschild geometry combined with a dipole magnetic field, fixing the equatorial plane of the background at the symmetry plane of the field. The line element of the vacuum Schwarzschild spacetime in the standard Schwarzschild coordinates (t,r,θ,φ) takes the form

(4) $$ds^{2}=-f\left(r\right)dt^{2}+f{\left(r\right)}^{-1}dr^{2}+r^{2}\left(d\theta^{2}+\sin^{2}\theta d\varphi^{2}\right),$$

with the lapse function

(5) $$f\left(r\right)=1-\frac{2M}{r}.$$

The mass of the extremely compact object is *M*. The dipole magnetic field of the considered extremely compact object is assumed to be generated by a current loop located inside or on the surface of a black hole mimicker with radius a=2M(1+δ), where δ≪1 (or inside an extremely compact Buchdahl star [30]).

The four-vector electromagnetic potential $A^{\mu }$ of the outer part of the dipole field solution (r>a) has the form $A_{\mu }=\left(0,\;0,\;0,\;A_{\varphi }\right)$, where

(6) $$\begin{array}{ccc} A_{\varphi } & = & -B\;h_{1}\left(r,\theta \right)=-B\;h\left(r\right)\;g_{\varphi \varphi }\left(r,\theta \right), \end{array}$$

with

(7) $$\begin{array}{ccc} & & h\left(r\right)=\ln\left(1-\frac{2M}{r}\right)+\frac{2M}{r}\left(1+\frac{M}{r}\right). \end{array}$$

The electromagnetic potential has the same form if it is generated by an internal magnetic field of the central object (an NS or a BH mimicker) [16,17] or an electric current loop located outside the object [14,15]. The parameter B, which determines the magnetic field intensity, has the form

(8) $$B=\frac{3\mu }{8M^{3}};$$

μ denotes the magnetic dipole moment. The Maxwell tensor, $F_{\mu \nu }=A_{\nu ,\mu }-A_{\mu ,\nu }$, has two non-zero components:

(9) $$\begin{array}{ccc} F_{\varphi r} & = & \frac{\partial A_{\varphi }}{\partial r}=B^{\theta }\\ & = & 2B\sin^{2}\theta \left(\frac{2M(r-M)}{2M-r}-r\ln\frac{r-2M}{r}\right) \end{array}$$

and

(10) $$\begin{array}{ccc} F_{\theta \varphi } & = & \frac{\partial A_{\varphi }}{\partial \theta }=B^{r}\\ & = & -B\sin\left(2\theta \right)\left[r^{2}\ln\frac{r-2M}{r}+2M\left(M+r\right)\right] \end{array}$$

In the equatorial plane (θ=π/2), only $B^{r}$ vanishes. On the symmetry axis (θ=0), both $B^{r}$ and $B^{\theta }$ vanish; therefore, no equilibrium points of charged particles can exist on the symmetry axis. The equipotential surfaces and the radial and θ profiles of both components of the Maxwell tensor of the dipole magnetic field are illustrated in [13].

In order to introduce the magnetic parameter, similarly to the case of the Schwarzschild black hole immersed in an asymptotically uniform magnetic field, we express the magnetic dipole moment in terms of the magnetic field intensity $B=B^{\theta }$ measured in the equatorial plane at the surface of the extremely compact object R=a=2M+δ. Using the magnetic field intensity $B^{\hat{\theta }}$ measured by local static observers at the surface of the considered object, the magnetic dipole moment can be expressed as [18]

(11) $$\mu =\frac{4M^{3}R^{3/2}\sqrt{R-2M}}{6M(R-M)+3R(R-2M)\ln f(R)}B^{\hat{\theta }}.$$

The electromagnetic potential, determined using Equation (6), can be thus given by the surface magnetic field intensity $B^{\hat{\theta }}\left(R,\theta =\pi /2\right)$.

## 3. Motion of Charged Test Particles and Its Effective Potential

The Lorentz equation governing the charged test particle motion has the form

(12) $$\frac{du^{\mu }}{d\tau }+\Gamma_{\alpha \beta }^{\mu }u^{\alpha }u^{\beta }=\frac{q}{m}F_{\alpha }^{\mu }u^{\alpha },$$

where $u^{\mu }$ denotes the four-velocity of the particle with mass *m* and charge *q*, normalized by the condition $u_{\alpha }u^{\alpha }=-1$; τ is the proper time of the particle.

The particle motion can be effectively described in the Hamiltonian formalism [12,31]. For the charged particle motion, the Hamiltonian *H* has the form

(13) $$H=\frac{1}{2}g^{\alpha \beta }\left(\pi_{\alpha }-qA_{\alpha }\right)\left(\pi_{\beta }-qA_{\beta }\right)+\frac{m^{2}}{2},$$

where the canonical four-momentum is given by $\pi_{\mu }=mu_{\mu }+qA_{\mu }$. The Hamilton equations of motion are used in the standard form

(14) $$\frac{dx^{\mu }}{d\zeta }=\frac{\partial H}{\partial \pi_{\mu }},\;\frac{d\pi_{\mu }}{d\zeta }=-\frac{\partial H}{\partial x^{\mu }},$$

where ζ=τ/m. Due to the symmetries of the Schwarzschild spacetime (4) and the dipole magnetic field (6), two conserved quantities of particle motion exist—the covariant-specific energy and the covariant-specific axial angular momentum

(15) $$E=\frac{E}{m}=-\frac{\pi_{t}}{m}=-g_{tt}u^{t},\;L=\frac{L}{m}=\frac{\pi_{\varphi }}{m}=g_{\varphi \varphi }u^{\varphi }+\bar{q}A\varphi ,$$

where $\bar{q}=q/m$ is the specific charge of the particle. The Hamiltonian thus can be separated into two parts,

(16) $$\begin{array}{ccc} \frac{H}{m^{2}} & = & H_{D}+H_{P}, \end{array}$$

where the dynamical and potential parts, $H_{D}$ and $H_{P}$, respectively, are expressed in the following way:

(17) $$\begin{array}{ccc} & & H_{D}=\frac{1}{2}\left(g^{rr}u_{r}^{2}+g^{\theta \theta }u_{\theta }^{2}\right), \end{array}$$

(18) $$\begin{array}{ccc} & & H_{P}=\frac{1}{2}\left[g^{tt}E^{2}+g^{\varphi \varphi }{\left(L-\bar{q}A_{\varphi }\right)}^{2}+1\right]. \end{array}$$

The availability regions of the test particle motion in the r−θ plane, given by the condition $H_{P}=0$, can be found conveniently by introducing a 2D effective potential related to the particle covariant energy. This allows for the straightforward determination of the circular orbits and the epicyclic oscillations of a harmonic nature around the stable circular orbits. Generally, the motion in the considered background is of a chaotic nature [4]. The effective potential $V_{\mathrm{eff}}\left(r,\theta ;L,b\right)$, determining the particle motion dependent on the motion constants and the background parameters, reads [13]

(19) $$\begin{array}{ccc} & & V_{\mathrm{eff}}\left(r,\theta ;L,b\right)\equiv -g_{tt}\left[g^{\varphi \varphi }{\left(L-\bar{q}A_{\varphi }\right)}^{2}+1\right]\\ & & =\left(1-\frac{2M}{r}\right)\left[{\left(\frac{L}{r\sin\theta }-b\;h\left(r\right)\;r\sin\theta \right)}^{2}+1\right]. \end{array}$$

The effective potential is thus governed by the specific axial component of angular momentum L and the “magnetic parameter” *b*, determining the intensity of the particle interaction with the given combined gravitational and electromagnetic fields,

(20) $$b=\bar{q}B=\frac{q}{m}\frac{3\mu }{8M^{3}};$$

the magnetic parameter can be taken in terms of the surface intensity of the magnetic field—this parameter corresponds to the parameter $b_{u}$ applied in the case of uniform magnetic fields. The Maxwell tensor component $F_{r\varphi }$, giving the radial component of the Lorentz force $F^{r}=F_{\varphi }^{r}u^{\varphi }$ in the equatorial plane, implies that a positive (negative) value of the magnetic parameter *b* corresponds to magnetic attraction (repulsion) with an inward (outward)-directed electromagnetic Lorentz force, if we restrict this to the corotating motion with $u^{\varphi }>0$. For such motion, the Keplerian angular velocity $\Omega_{\varphi }=u^{\varphi }/u^{t}>0$ corresponds to corotation relative to static observers at infinity. The turning points of a particle’s motion are determined by the condition

(21) $$E^{2}=V_{\mathrm{eff}}\left(r,\theta ;L,b\right).$$

The effective potential is symmetric relative to the simultaneous change in sign of the angular momentum L and the magnetic parameter *b*. It is thus natural to focus on the family of orbits with L>0, which are usually corotating, and the cases of magnetic repulsion (b<0) and magnetic attraction (b>0) [12].

However, as will be shown in the following section, for extremely compact objects mimicking black holes, an important part of the equatorial circular motion under magnetic repulsion corresponds to the case of particles with b<0, L<0, and $\Omega_{\varphi }>0$.

In the following, for simplicity, we use M=1. We thus express the radius, time, and other quantities in units of the mass parameter *M*, i.e., the gravitational radius of the central compact object.

## 4. Circular Orbits

The circular orbits of charged particles orbiting in the Schwarzschild spacetime endowed with a dipole magnetic field were studied in detail in [13], where the motion was studied in the region of r>3, being focused on the case of neutron stars. Here, we briefly summarize the results of a detailed study related to the region of r>3 [13] and extend them to the region 2<r<3, which can be relevant in the case of black holes and their mimickers. We focus on the aspects of the circular motion relevant to the behavior of ionized Keplerian disks, namely marginal stability against perturbations orthogonal to the equatorial plane, which allows the existence of off-equatorial circular orbits related to motion in belts.

Circular orbits are determined by the simultaneous vanishing of the effective potential derivatives with respect to the coordinates *r* and θ: $\partial_{r}V_{\mathrm{eff}}=0$ and $\partial_{\theta }V_{\mathrm{eff}}=0$. The orbital angular velocity of the circular motion related to the proper time of the orbiting particle is determined by the relation

(22) $$\begin{array}{c} \omega_{\varphi }\left(r,\theta \right)=\frac{d\varphi }{d\tau }=u^{\varphi }=Lg^{\varphi \varphi }\left(r,\theta \right)-bh\left(r,\theta \right). \end{array}$$

### 4.1. Equatorial Circular Orbits

For motion in the equatorial plane (θ=π/2), there is $\partial_{\theta }V_{\mathrm{eff}}=0$, which is independent of the value of *b*. Therefore, equatorial circular orbits exist for any *b*, determined by the condition $\partial_{r}V_{\mathrm{eff}}=0$, which implies the radial profile of the specific angular moments of these orbits in the form

(23) $$\begin{array}{ccc} & & L_{c}^{\pm }=\frac{b}{r-3}\left[r^{2}\;\mathrm{Ln}(r)+2r+6\right]\pm r\frac{▵(r,b)}{r-3}, \end{array}$$

where

(24) $$▵(r,b)=\sqrt{b^{2}{\left[2(r-1)+(r-2)r\;\mathrm{Ln}(r)\right]}^{2}+r-3}$$

and

(25) $$\mathrm{Ln}(r)=\ln(\frac{r-2}{r})$$

The radial profile of the specific energy of the equatorial circular orbits, determined by the condition $E_{c}^{2}=V_{\mathrm{eff}}\left(r,L=L_{c}\right)$, then reads

$$\begin{array}{c} E_{c}=r^{-1}\sqrt{1-\frac{2}{r}}\{-br^{2}\pm [r\frac{▵(r,b)}{r-3}+\\ b+\frac{br^{2}\;\mathrm{Ln}(r)+2br+6b}{r-3}-r^{2}\;\mathrm{Ln}(r)-2\left(r+1\right){]}^{2}{\}}^{\frac{1}{2}}. \end{array}$$

Note that the presented solutions for equatorial circular orbits have symmetry against a simultaneous change in the sign of the parameter *b* and the ± branches of the solutions for L and E.

The photon circular orbit, related to the Schwarzschild geometry, is located at r=3M and represents a limit on the existence of circular orbits of uncharged particles. The reality condition

(26) $${\left[2b(r-1)+b(r-2)r\;\mathrm{Ln}(r)\right]}^{2}+r-3\geq 0$$

governs the existence of equatorial circular orbits at radii 2<r<3; there is no restriction on the existence of circular equatorial orbits of charged particles at radii r≥3. The existence of equatorial circular orbits is limited by the critical function of the magnetic parameter *b* given by the relation

(27) $$b_{\mathrm{cr}}^{\pm }\left(r\right)=\pm \frac{3-r}{2(r-1)+(r-2)\mathrm{Ln}(r)}.$$

**Figure 1:**
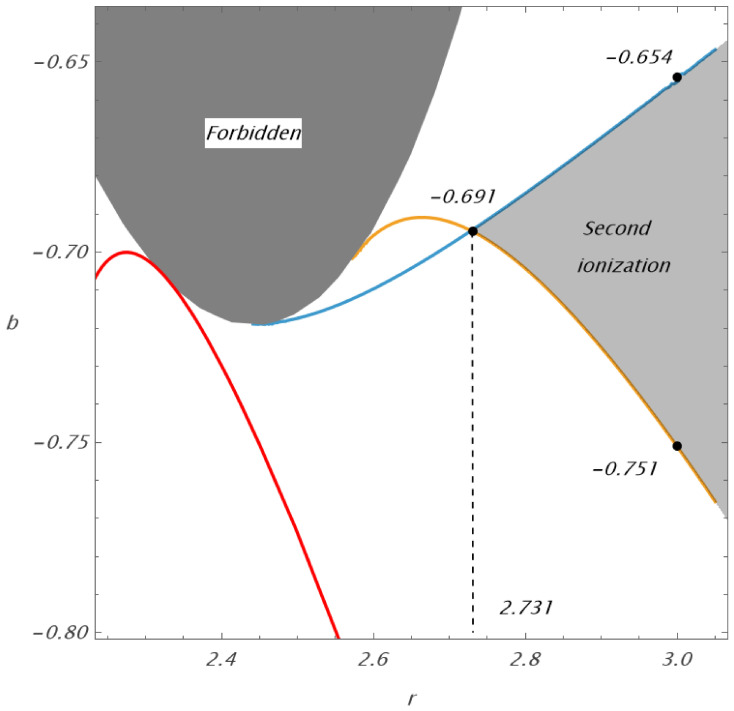
Curves of vanishing second derivatives of the effective potential in the equatorial plane $V_{\mathrm{eff}}\left(r,\theta =\pi /2\right)$ as a function of the radial coordinate *r* and parameter *b*, giving the functions $b_{\mathrm{rISCO}}\left(r\right)$ and $b_{\theta ISCO}\left(r\right)$. The red curves reflect $b_{L=0}\left(r\right)$. The function $b_{\mathrm{cr}}^{\pm }$ limits the black region, forbidden for circular motion. Curves of vanishing second derivatives of the effective potential in the equatorial plane $V_{\mathrm{eff}}\left(r,\theta =\pi /2\right)$ as a function of the radial coordinate *r* and parameter *b*, giving the functions $b_{\mathrm{rISCO}}\left(r\right)$ (blue) and $b_{\theta ISCO}\left(r\right)$ (orange). The red curves reflect $b_{L=0}\left(r\right)$. The function $b_{\mathrm{cr}}^{\pm }$ limits the black region, forbidden for circular motion.

This critical function is represented in Figure 1. The limit points of the critical function $b_{\mathrm{cr}}\left(r\right)$ are at $r_{\mathrm{cr}}≐2.441$, $b_{\mathrm{cr}(e)}≐\pm 0.719$.

We can see that, for small values of the magnetic parameter *b*, the corresponding equatorial circular orbits close to the geodesic circular photon orbit can cross the radius r=3, reaching a minimal radius $r_{min}>r_{cr}=2.441$. An increasing magnitude of *b*, either positive or negative, causes the appearance of a second region of circular orbits at $2<r<r_{max}<r_{cr}=2.441$. With increasing *b*, both regions merge, and circular orbits are allowed at r>2M if |b|>0.719.

Concerning the behavior of ionized Keplerian disks—and in astrophysical situations in general—the locations of marginally stable circular orbits are crucial. For stable circular orbits, the conditions $d^{2}V_{\mathrm{eff}}/dr^{2}>0$ and $d^{2}V_{\mathrm{eff}}/d\theta^{2}>0$ have to be satisfied simultaneously. Marginally stable circular orbits are determined by the conditions $d^{2}V_{\mathrm{eff}}/dr^{2}=0$ and $d^{2}V_{\mathrm{eff}}/d\theta^{2}=0$. Therefore, we consider two characteristic radial functions of the parameter *b* governing marginal stable circular orbits, assuming a family of corotating orbits with $u^{\varphi }>0$.

The characteristic function of circular orbits that are marginally stable against radial perturbations, denoted by $b_{\mathrm{rISCO}}\left(r\right)$, can be determined only numerically, as it is implicitly determined by a complex relation introduced in [13]. The characteristic function of circular orbits that are marginally stable against latitudinal perturbations, denoted by $b_{\theta ISCO}\left(r\right)$, is explicitly determined by the relation

(28) $$\begin{array}{ccc} b_{\theta ISCO}\left(r\right) & = & -\frac{r}{2}[-r^{4}{\mathrm{Ln}}^{2}\left(r\right)-4r^{3}\;\mathrm{Ln}(r)\\ & & -4r^{2}\left(1+2r^{2}\;\mathrm{Ln}(r)\right)-16r-12{]}^{-1/2}. \end{array}$$

Both functions $b_{\mathrm{rISCO}}\left(r\right)$, $b_{\theta ISCO}\left(r\right)$ are illustrated in Figure 1 and are related to the reality critical function $b_{\mathrm{cr}}^{\pm }$. The region of stability relative to radial perturbations is located under the curve $b_{\mathrm{rISCO}}\left(r\right)$, while the region of stability relative to latitudinal perturbations is located above the curve $b_{\theta ISCO}\left(r\right)$ [13]. (The functions are presented in the region of 2<r<3; a more extended view of these functions can be found in [13].) We add also the characteristic function of the magnetic parameter, defined as

(29) $$b_{L=0}\left(r\right)=\pm \frac{r}{\sqrt{-4\left(1+r\right)\left(3+r^{2}\right)-4\left(r^{2}+r^{4}\right)\mathrm{Ln}(r)r-\left(r-1\right)r^{4}{\mathrm{Ln}(r)}^{2}r}}$$

which determines the positions of corotating equatorial circular orbits with the specific axial angular momentum L=0. Under the critical function $b_{L=0}\left(r\right)$, the corotating orbits have L<0; such equatorial circular orbits are unstable against latitudinal perturbations.

We can see that the critical functions $b_{\mathrm{rISCO}}\left(r\right)$, $b_{\theta ISCO}\left(r\right)$ have a common critical point at the radius $r_{c(ms)}=2.731$ for the critical magnetic parameter $b_{c(ms)}=-0.691$. The radius of the radially (vertically) marginally stable orbit increases (decreases) from the critical point. Stable circular orbits located under the photon circular geodesic (at $r_{ph}=3$) can exist for a magnetic parameter within the interval −0.751<b<−0.654. Under a marginally stable circular geodesic (located at $r_{ms}=6$), charged particle circular orbits can exist for magnetic parameters within the interval −2.105<b<0. For magnetic attraction, b>0, and magnetic repulsion with magnetic parameter b<−2.105, stable circular orbits exist only above the marginally stable circular geodesic—these limits are crucial for the behavior of ionized Keplerian disks. In the case of magnetic attraction, the unstable region above r=6 falls directly onto the central object. In the case of magnetic repulsion, the disk region corresponding to instability against latitudinal perturbations enters the motion in belts centered around off-equatorial circular orbits.

### 4.2. Off-Equatorial Motion

Off-equatorial circular orbits are crucial for the fates of ionized Keplerian disks under magnetic repulsion. In contrast to the case of equatorial orbits, off-equatorial circular orbits have to be determined through the simultaneous solution of equations $dV_{\mathrm{eff}}/dr=0$ and $dV_{\mathrm{eff}}/d\theta =0$. Therefore, the magnetic parameter *b* and the motion constant L must be related to the coordinate θ, determining the circular orbit’s position, along with the radial coordinate—see [13].

An off-equatorial circular orbit fixed at a given point on the r−θ plane is possible for a charged particle with parameter *b* determined by the relation

(30) $$b_{\mathrm{Coff}}\left(r,\theta \right)=\pm \frac{-r\csc\theta }{2\sqrt{r^{2}\;\mathrm{Ln}(r)+2r+2}\sqrt{r^{2}\;\mathrm{Ln}(r)+2r+6}}.$$

The specific angular momentum $L_{\mathrm{Coff}}$ can then be determined as

(31) $$\begin{array}{ccc} L_{\mathrm{Coff}}\left(r,\theta \right) & = & \pm \frac{-r\sqrt{r^{2}\;\mathrm{Ln}(r)+2r+2}\sin\theta }{2\sqrt{r^{2}\;\mathrm{Ln}(r)+2r+6}}. \end{array}$$

The specific energy $E_{\mathrm{Coff}}$ of the circular off-equatorial orbit has to be calculated for an appropriate combination of the $b_{\mathrm{Coff}}\left(r,\theta \right)$ and $L_{\mathrm{Coff}}\left(r,\theta \right)$ functions. It should be noted that solutions with a positive value must be considered, as we focus on the astrophysically relevant positive root states [31,32]

(32) $$\begin{array}{ccc} E_{\mathrm{Coff}}\left(r\right) & = & 2\sqrt{\frac{r-2}{r\left(r^{2}\mathrm{Ln}(r)+2r+6\right)}}. \end{array}$$

Note that the specific energy $E_{\mathrm{Coff}}$ of off-equatorial circular orbits depends only on the radial coordinate; the additional latitudinal coordinate of the orbit implies the magnetic parameter of the orbit (charge of the particle) and the specific angular momentum $L_{\mathrm{Coff}}$. It must also be noted that off-equatorial circular orbits are stable (unstable) against perturbations at r>3 (2<r<3), as demonstrated in [13]. For this reason, the motion of charged particles in belts restricted to regions in proximity to stable off-equatorial circular orbits is possible.

For the family of corotating orbits (with $u^{\varphi }>0$ and $\omega_{\varphi }>0$), off-equatorial orbits can exist only for charged particles with b<−0.691, i.e., for magnetic repulsion—particles under magnetic attraction (b>0) have to follow counter-rotating off-equatorial orbits with $\omega_{\varphi }<0$. Off-equatorial corotating circular orbits start at the radius of the equatorial circular orbit that is marginally stable relative to latitudinal perturbations [13]. Therefore, their existence is allowed only for magnetic parameters

(33) $$b\leq b_{tisco(max)}=b_{c(ms)}=-0.691.$$

**Figure 2:**
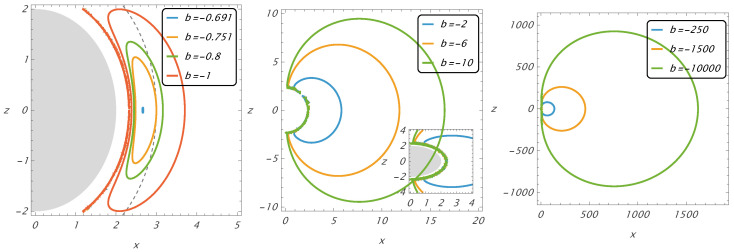
The positions of off-equatorial circular orbits that are stable at r>3.

For $b=b_{tisco(max)}≐-0.691$, off-equatorial circular orbits are reduced to one point located at the equatorial plane at the radius $r_{tisco(max)}=2.663$—see also Figure 2, giving the behavior of the function $b_{\mathrm{Coff}}\left(r,\theta \right)$ at small radii.

The function $b_{\mathrm{Coff}}\left(r,\theta \right)$, which is symmetric relative to the equatorial plane, enables us to directly determine the positions of circular orbits located off the equatorial plane. Possible positions for off-equatorial circular orbits, as given by Equation (30), are represented for selected values of the magnetic parameter in Figure 2, where the Cartesian coordinates x,z are used instead of the spherical coordinates r,θ. The off-equatorial circular orbits approach r=2 along the symmetry axis of the magnetic field.

The sequence of the off-equatorial circular orbit starts at the equatorial plane at $r_{\theta ISCO}$, which can be observed for b≪−1 approximated by the relation $r_{\theta ISCO}=4\sqrt[{3}]{\frac{2}{3}}{\left(-b\right)}^{2/3}$ [13], being limited from below by the equatorial critical radius $r_{cr}\left(-b\to \infty \right)=2.323$.

For sufficiently large magnitudes of *b*, the functions $b_{\mathrm{Coff}}\left(r,\theta \right)=const$ resemble oblate ellipsoids that are strongly deformed in close proximity to r=2. The functions start very far from the r=2 surface near the equatorial plane (for θ∼π/2) at $r_{\theta ISCO}$, but they rapidly approach the surface r=2 as they approach the symmetry axis (θ→0). In the region of intermediate values of θ, the variation in *x* is rapid, while *z* varies slowly. With decreasing magnitudes of *b*, the extension of the off-equatorial orbits decreases as the radius $r_{\theta ISCO}$ decreases. For sufficiently low magnitudes of *b*, the functions $b_{\mathrm{Coff}}\left(r,\theta \right)=const$ representing the family of off-equatorial circular orbits are extended in the vicinity of r=2, and they resemble prolate ellipsoids that are reduced to a point on the equatorial plane in the limiting case of $b=b_{tisco(max)}=-0.691$.

As demonstrated in [13], all off-equatorial circular orbits are stable in the region of r>3 and unstable in the region of 2<r<3. In the region of stability, the energies of these orbits are bound, having $E_{Coff}<1$.

The circular orbits located “in” and “off” the equatorial plane correspond to minima of the effective potential, related to a given test particle with parameter *b*, a related specific angular momentum $L_{c}$, and the specific energy $E_{c}$. If the specific energy of a particle with fixed values of *b* and $L_{c}$ is slightly increased, the particle follows a harmonic epicyclic oscillatory motion around the minimum, while a further increase in its specific energy leads to a transformation into a chaotic motion around the circular orbits or in extended belts centered around these circular orbits. These properties of the circular orbits of charged test particles can be reflected in the behavior of ionized Keplerian disks, especially regarding the transition between regular and chaotic motion, with strong differences in motion under repulsive and attractive magnetic forces.

## 5. Ionized Keplerian Disks

We consider Keplerian disks orbiting a compact object represented by the Schwarzschild spacetime describing the gravitational field of a black hole (or, alternatively, its mimicker or a neutron star), which are ionized and influenced by a dipole magnetic field related to this compact object. Alternatively, to study the vicinities of these extremely compact objects, we consider also the fate of an equatorial disk of charged particles undergoing an increased level of ionization, reflected by an increase in the magnitude of the magnetic parameter *b*. Then, we compare the results obtained for the dipole magnetic field with results regarding the influence of an external, asymptotically uniform magnetic field, which can be relevant, for example, in the case of supermassive black holes located in active galactic nuclei. (The asymptotically uniform magnetic field can offer a good approximation of the parabolic fields considered in models of general-relativistic magnetohydrodynamic accretion processes in active galactic nuclei [10,33,34,35]).

In our work, we are interested in the interplay of the gravitational and electromagnetic forces in the vicinity of the central compact object. We thus consider the region of extension $r\leq 10^{2}r_{g}$, focusing, in special cases, on the immediate vicinity of the central object with $r<10r_{g}$. Such a restriction could correspond to accretion disks created by tidal effects of the central compact object onto some orbiting planet, star, or other satellite orbiting at a small distance from the central compact object [36,37,38]. Therefore, the inclination of the disk relative to the symmetry axis of the magnetic field can be considered arbitrary. (For simplicity, we do not consider here the possible effects of alignment torque, as it is beyond the scope of the present paper, requiring the specification of the astrophysical environment.) Of course, we consider also the central parts of extended Keplerian disks evolved around the central compact object in its equatorial plane.

### 5.1. Keplerian Disks

Keplerian disks are governed by the circular geodesics of the spacetime. In the case of the spherically symmetric Schwarzschild spacetime, the Keplerian disk can be located in any of the central planes of the spacetime. Therefore, there can be equatorial Keplerian disks located in the central plane orthogonal to the magnetic dipole axis or Keplerian disks inclined to the axis. We consider both cases.

The radial profiles of the specific energy and specific angular momentum of the circular geodesics are given by the relations [31]

(34) $$E\left(r\right)=\frac{1-\frac{2}{r}}{\sqrt{1-\frac{3}{r}}},\;L\left(r\right)=r\sqrt{\frac{1}{r-3M}}.$$

The marginally stable circular orbit of the Schwarzschild spacetime is located at $r_{ms}=6$, giving the inner edge of the Keplerian disk.

Note that, due to the ionization process, the particle-specific axial angular momentum is modified due to the action of the dipole magnetic field, while the particle-specific energy remains unchanged—see Equation (15). Moreover, an energy gain is not possible, in contrast to the case of rotating compact objects [4].

### 5.2. Ionization Process

The combined gravomagnetic field of magnetized black hole mimickers is represented by the magnetic parameter *b*. The role of the parameter *b* in demonstrating the three regimes of particle motion can be illustrated by the fate of a Keplerian disk after the ionization of its inner part. We assume a Keplerian disk constituted by electrically neutral matter following the circular geodesics of the Schwarzschild spacetime, with the symmetry plane of the Keplerian disk being identical (or almost identical) to the symmetry plane of the dipole magnetic field, or in a highly inclined Keplerian disk. The ionization of the neutral matter can be realized due to the irradiation of electrically neutral atoms by photons of sufficiently high energy or by the spontaneous decay of free neutrons into protons and electrons [4], representing a magnetic Penrose process [8,39,40,41]. Such a process is characterized by charge conservation and canonical momentum conservation. If the first particle is electrically neutral ($q_{1}=0=q_{2}+q_{3}$), the canonical momentum conservation can be given in the form

(35) $$\begin{array}{c} p_{\alpha (1)}=p_{\alpha (2)}+q_{2}A_{\alpha }+p_{\alpha (3)}+q_{3}A_{\alpha }=p_{\alpha (2)}+p_{\alpha (3)}. \end{array}$$

In the ionization cases considered in the present paper, the created charged particles, i.e., proton (ion) versus electron, have a rest mass ratio $m_{2}/m_{3}\gg 1$—the more massive particle takes almost all the momentum of the original neutral particle, while the influence of the lighter particle can be neglected, implying [4,9,10,12,42]

(36) $$\begin{array}{c} p_{\alpha (1)}∼p_{\alpha (2)}\gg p_{\alpha (3)}. \end{array}$$

In the following, we study only the fates of the more massive particles (proton, ions).

As the dipole magnetic field considered in the present paper is determined only by the axial component of the electromagnetic potential $A_{\varphi }$, the energy $E=-p_{t}$ of the created particles is conserved in the ionization process, and these particles cannot escape to infinity as the neutral particles of the Keplerian disk follow stable circular geodesics with E<1 [12]. (Escaping and highly accelerated charged particles can be generated in the ionization process around the Kerr black holes because of the non-zero $A_{t}$ component of the electromagnetic potential caused by the rotation of the spacetime [8,41]).

The initial condition for the particles of a slightly inclined ionized Keplerian disk can be given in simple form. The position $x^{\alpha }$ and 4–velocity $u_{\alpha }$ are determined as

(37) $$\left(t,r,\theta ,\varphi \right)=\left(0,r_{0},\theta_{0},0\right),$$

(38) $$\left(u_{t},u_{r},u_{\theta },u_{\varphi }\right)=\left(E,0,0,L\right).$$

The specific energy and specific axial component of the angular momentum of an uncharged particle following an inclined circular geodesic are given as [9]

(39) $$E_{1}=\frac{r_{0}-2}{\sqrt{r_{0}^{2}-3r_{0}}},$$

(40) $$L_{1}=\frac{r_{0}\sin\theta_{0}}{\sqrt{r_{0}-3}}.$$

After the ionization process, the second, much more massive particle has the specific energy

(41) $$E_{2}=E_{1},$$

and the specific angular momentum

(42) $$L_{2}=L_{1}+\frac{q}{m}A_{\varphi }\left(r_{0},\theta_{0}\right).$$

The electromagnetic part of the conserved specific angular momentum $L_{2}\left(r_{0},\theta_{0}\right)$ can be expressed as in the definition of the effective potential in terms of the motion constant *b*—see Equation (20).

The charged particles of the ionized Keplerian disk orbiting in the Schwarzschild background with a dipole magnetic field can be captured by the central object, oscillate in the epicyclic motion around the original circular geodesic, or enter a state of chaotic bound motion or the magnetic regime of the motion. The escape of such a particle to infinity would be possible only due to an additional kick (see [43]) or due to rotational effects related to the central object [9]. As the particles of the Keplerian disk are bound, having E<1, and their energy is not increasing due to the ionization process, where only the axial angular momentum is changed, the ionized particles remain bound. In the case of magnetic repulsion, they can concentrate their motion around off-equatorial circular orbits having $E_{Coff}<1$, forming structures resembling belts. We can thus expect to observe charged particle belts around the compact object.

### 5.3. Astrophysical Relevance, Scaling Symmetry, and Approximate Self-Similarity of Charged Particle Motion

In order to clearly illustrate the physical substance of the phenomena related to charged particle dynamics, representative values of the parameters characterizing the considered phenomena are chosen. However, such representative values, enabling efficient calculations, may differ significantly from those corresponding to realistic astrophysical situations related to elementary particles. In our study, for the purpose of numerical calculation, we consider moderate values of the magnetic parameter, |b|∼1, reaching up to $\left|b\right|∼10^{2}$. Meanwhile, in situations related to magnetized black holes (their mimickers) or neutron stars, under the consideration of ions, protons, and electrons, extreme values of |b|≫1 are more realistic. In Table 1, we give the magnitude of the Lorentz force acting on protons and moving in a magnetic field of intensity *B*, related to the gravitational force assumed for a black hole of mass $M=10M_{⊙}$, giving the magnetic parameter |b|. We can see that, for the motion of protons (and ions), the magnitude of the magnetic parameter |b|∼1 can correspond to the motion in the vicinity of a black hole or black hole mimicker, where the magnetic field is weak (not exceeding 10G). However, such values are irrelevant regarding motion around strongly magnetized black holes and neutron stars with a magnetic field intensity in the interval of $10^{4}G<B<10^{15}G$, where |b|≫1.

However, in the case of a dipole magnetic field, the equations governing charged particle motion can be transformed into the form

(43) $$\frac{d\varphi }{d(\tau b)}=\frac{L}{b}g^{\varphi \varphi }\left(r,\theta \right)-h_{1}\left(r,\theta \right),$$

and

(44) $$\begin{array}{cc} \frac{H}{m^{2}}= & \frac{1}{2}[g_{rr}{\left(\frac{dr}{d(\tau b)}\right)}^{2}+g_{\theta \theta }{\left(\frac{d\theta }{d(\tau b)}\right)}^{2}+g^{tt}{\left(\frac{E}{b}\right)}^{2}+\\ & g^{\varphi \varphi }{\left(\frac{L}{b}-h_{1}\left(r,\theta \right)\right)}^{2}+\frac{1}{b^{2}}] \end{array}$$

and we observe the approximate scaling of the equations of motion, implying the approximate self-similarity of the motion and its trajectories. The scaling is precise in the case of azimuthal motion as it depends only on the fraction cl/b under the modified proper time τb. From the Hamiltonian, it follows that we have, in general, an approximate scaling as the motion depends on the fractions L/b and E/b under the modified time τb, assuming that the last term of the Hamiltonian, $1/b^{2}$, can be abandoned. Clearly, the approximation’s precision increases with increasing magnetic parameter values *b*. In fact, it is expected for b≫1—in such a case, the trajectories of charged particles can become indistinguishable under the rescaling of the motion parameters and time in the form

(45) $$L=L_{i}b,E=E_{i}b,\tau =\tau_{i}/b.$$

Therefore, for sufficiently high values of *b*, the motion has the same nature if the angular momentum and energy are appropriately increased, while the time speeds up correspondingly. We thus have strong evidence for the applicability of the results of numerically effectively tractable calculations to realistic but computationally demanding situations. In the case of ionized Keplerian disks, this self-similarity can be applied at least intuitively in the case of sufficiently large values of |b|, but it is not useful in the case of |b|∼1, where the role of the term $1/b^{2}$ in the Hamiltonian is significant. Furthermore, it must be stressed that the scaling self-similarity effect holds for any test magnetic field, i.e., a field that does not influence the geometry of the Schwarzschild spacetime.

In our study, we thus consider two regimes: one of weak magnetic fields represented by the interval 1<b<10, where the scaling effect is surely irrelevant, and one of strong magnetic fields represented by the interval 10<b<100, where the scaling effects become relevant. The latter can be treated as an approximate guide to the regime of extremely strong effects of the magnetic field, where *b* can exceed $b=10^{2}$. The chosen interval regarding the magnitude of the magnetic parameter enables an illustrative representation of the transitions between the magnetic, chaotic, and gravitational regimes of motion.

The influence of the magnetic parameter *b* on the ionized Keplerian disk’s fate is characterized by a sequence of figures constructed for Keplerian disks initially located exactly in the equatorial plane (the symmetry plane of the magnetic dipole), for disks slightly inclined to the symmetry plane, and for disks highly inclined to the symmetry plane. We consider the roles of both Lorentz repulsion and Lorentz attraction, taking characteristic values of the magnetic parameter *b* to demonstrate the possible roles of the dipole magnetic field depending on its intensity. We consider the cases of Lorentz repulsion and attraction separately.

As the inner edge of the uncharged Keplerian disk is located at $r_{ms}=6$, we are not able to start in close proximity to the compact object at $r∼r_{g}$. In order to test the interplay of the gravitational and electromagnetic forces, we apply the idea of secondary ionized charged disks, assuming the case of charged disks with a special value of b=−0.691, where the the stable circular orbits reach the value of $r_{ms(c)}=2.731$, located under the photon circular geodesic. In this special case, we can consider the effects of magnetic repulsion only, as secondary ionization can only increase the magnetic repulsion effect.

In order to demonstrate the interplay of the gravitational and magnetic forces, we use sequences of ionized disks with a magnetic parameter with small (1<|b|<10) and large (10<|b|<100) magnitudes in order to cover the transitions between the three regimes of charged particle motion, especially the interplay of the chaotic and magnetic regimes. We do not consider the case of |b|<1 as it corresponds here to a gravitational regime with small epicyclic oscillations around the circular geodesics [44,45,46].

Finally, we compare our results with those related to the fates of ionized Keplerian disks orbiting a Schwarzschild black hole immersed in an asymptotically uniform magnetic field.

### 5.4. Lorentz Repulsion

Lorentz repulsion, corresponding to magnetic parameters b<0, acts in the equatorial plane oppositely to the action of the gravitational force; however, outside the equatorial plane, the action of the dipole magnetic field is more complex and supports the chaotic nature of the motion.

Magnetic repulsion enables the existence of off-equatorial circular orbits, which should be reflected in the fates of ionized Keplerian disks through the creation of belts with chaotic charged particle motion.

In order to effectively illustrate the considered phenomena, we provide a global view of the ionized disks covering the region of ∼50$r_{g}$ and a detailed view of the inner region of ∼10$r_{g}$. Such extended regions enable us to cover least two regimes of motion—magnetic and chaotic. On the other hand, it should be noted that, for sufficiently large distances from the compact object, the ionized disk always enters the gravitational regime with small oscillations around the circular geodesics.

Note that, for the magnetic repulsion acting on the ionized disk, only the instability relative to vertical perturbations is relevant, causing the transformation of the appropriate region of the disk, located under the radius $r_{\theta ISCO}$, into chaotic motion captured in belts. Instability in the radial direction is impossible due to the magnetic repulsion.

#### 5.4.1. Equatorial Disks

**Figure A1:**
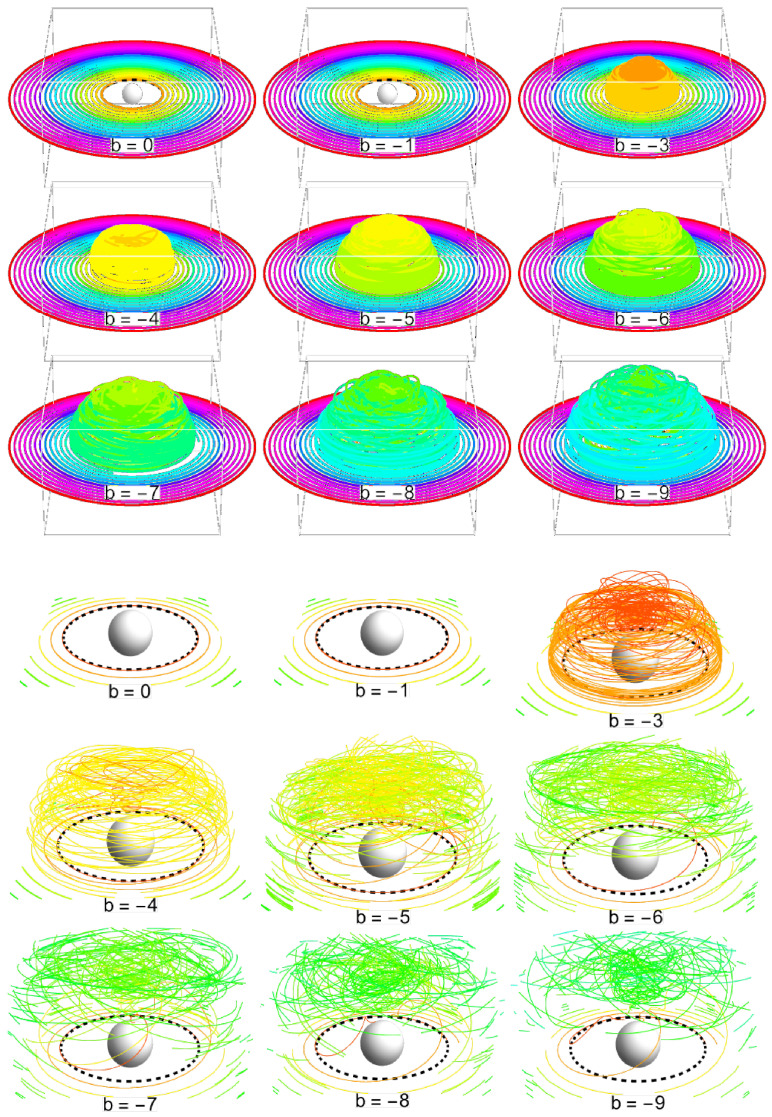
Keplerian disk on the equatorial plane with small magnitudes of negative parameter *b*. Upper panel: global view. Lower panel: zoom around the central object.

**Figure A2:**
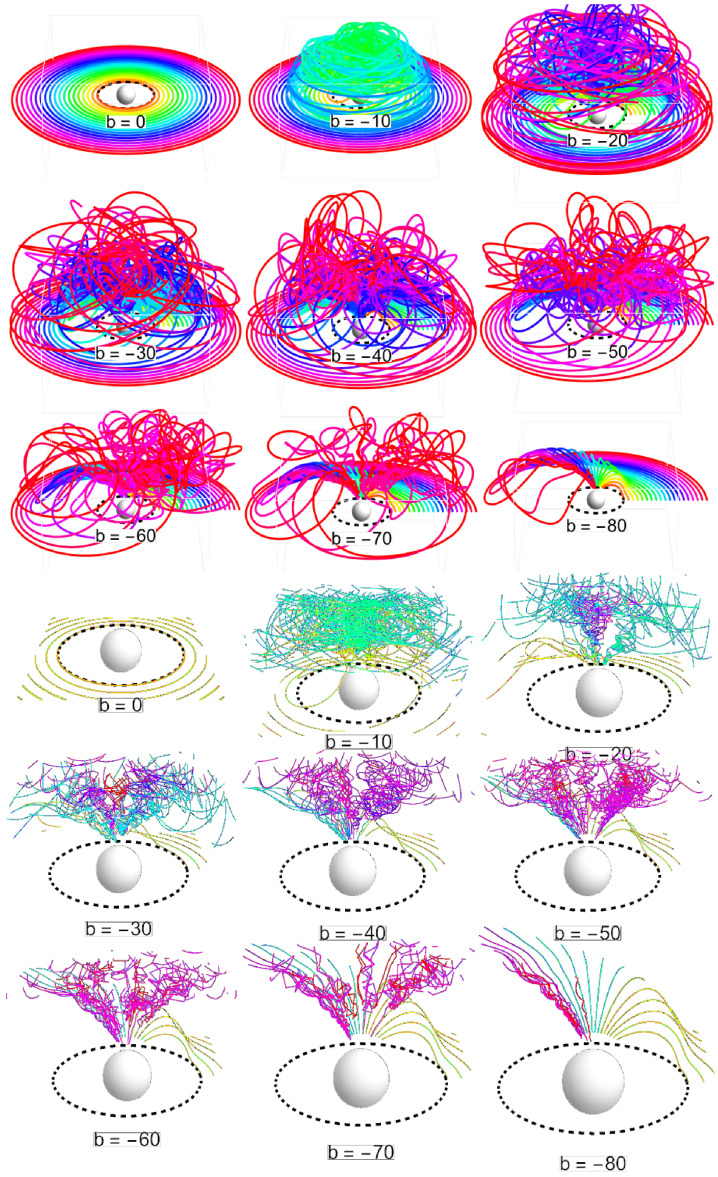
Keplerian disk on the equatorial plane with large magnitudes of negative parameter *b*. Upper panel: global view. Lower panel: zoom around the central object.

The fates of ionized equatorial Keplerian disks, where the initial conditions are related to the symmetry plane, enable a simple demonstration of the interplay of gravitational and magnetic forces. The resulting fate is represented in Figure A1 for small magnitudes of the magnetic parameter *b* and in Figure A2 for large magnitudes of this parameter; see Appendix A. This allows a clear illustration of the magnetic and chaotic regimes and their dependence on the magnetic parameter *b*.

As seen in the sequence for small magnitudes of *b*, for the smallest value of b=−1, the the gravitational regime prevails, and the chaotic regime emerges for b=−3, being limited to the immediate vicinity of the marginally stable circular geodesic. With increasing magnitudes of *b*, the region of chaotic motion is extended to larger radii, both in the equatorial plane and orthogonal to the plane. With increasing radii, the height of the motion decreases and it becomes more regular. The ionized disks thus resemble a “hill” constructed from shells at large distances from the center, while the interior consists entirely of chaotically moving particles.

The sequence of large magnitudes of *b* demonstrates a dramatic change. Starting from b∼−20, the innermost part of the Keplerian disk is fully under the magnetic regime, efficiently repelling the particles from this part of the Keplerian disk at large distances along regular trajectories. At larger distances, the ionized disk again resembles a “hill”. With increasing magnitudes of *b*, the region of particles repelled along regular trajectories increases, and the chaotic nature of the interior of the “hill-like” structures also increases—the inner parts of these structures exhibit the first indications of the chaotic motion centered around circular off-equatorial orbits. In the case of b=−80, almost the entire inner part of the ionized disk is repelled. With further increases in the magnitude of *b*, the repelled part increases and the chaotic “hill-like” structures are shifted to larger distances from the center.

#### 5.4.2. Slightly Inclined Disks

**Figure A3:**
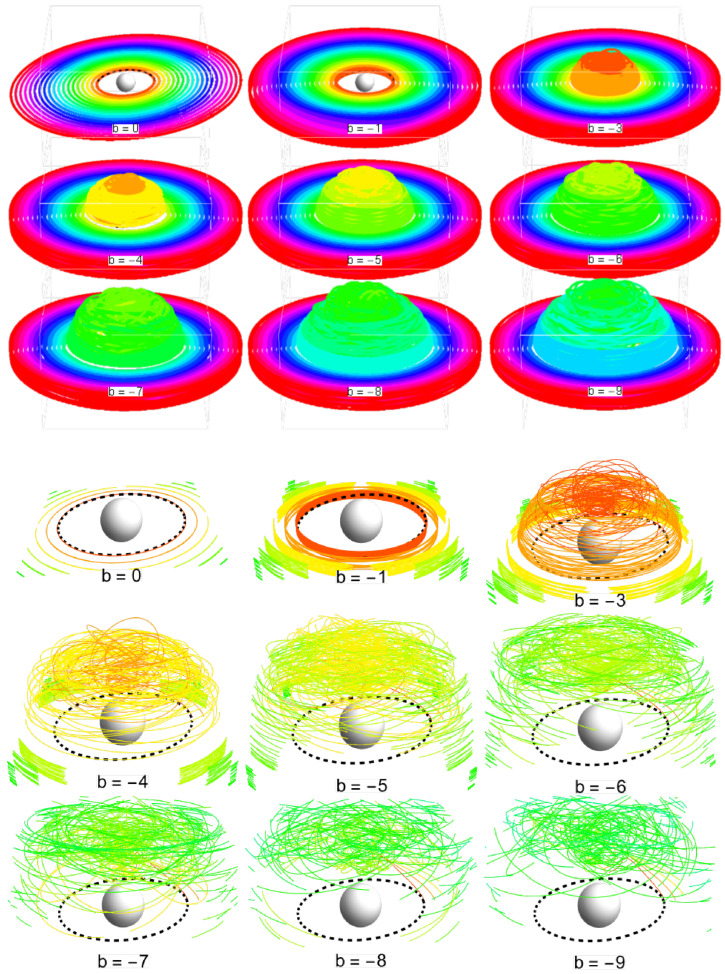
Keplerian disk slightly inclined to the equatorial plane (θ=1.5) with small magnitude of negative parameter *b*. Upper panel: global view. Lower panel: zoom around the central object.

**Figure A4:**
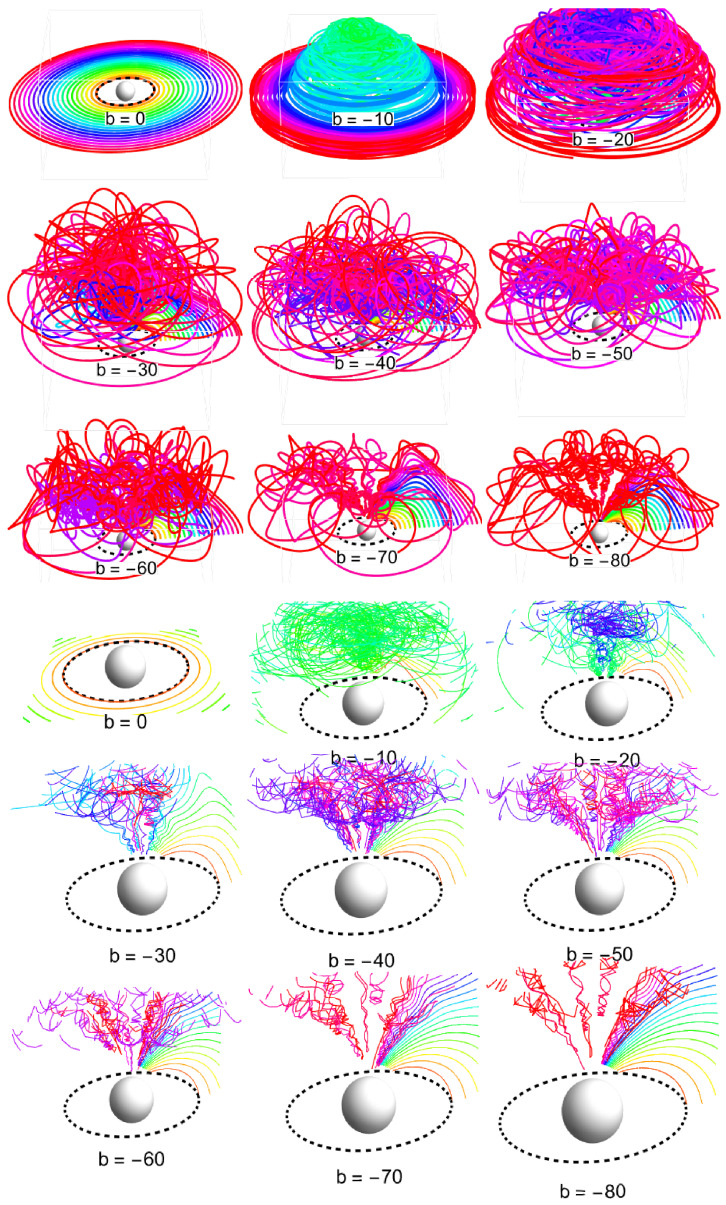
Keplerian disk slightly inclined to the equatorial plane (θ=1.5) with large magnitude of negative parameter *b*. Upper panel: global view. Lower panel: zoom around the central object.

Here, we consider how very small inclinations of the Keplerian disk relative to the symmetry plane can influence the fate of the ionized disk. The results are given in Figure A3 (Figure A4) for small (large) magnitudes of the magnetic parameter *b*; see Appendix A.

Both figures clearly demonstrate qualitative similarity to the previous case of the ionized equatorial disk, with one notable distinction—the stronger tendency toward chaotic motion. On the other hand, the case of b=−1 clearly illustrates the epicyclic oscillatory motion characteristic of the gravitational regime. Moreover, the tendency for chaotic motion in belts concentrated around off-equatorial circular orbits is well described (see, e.g., figures for magnetic parameters in the interval −4>b>−9).

#### 5.4.3. Highly Inclined Disks

**Figure A5:**
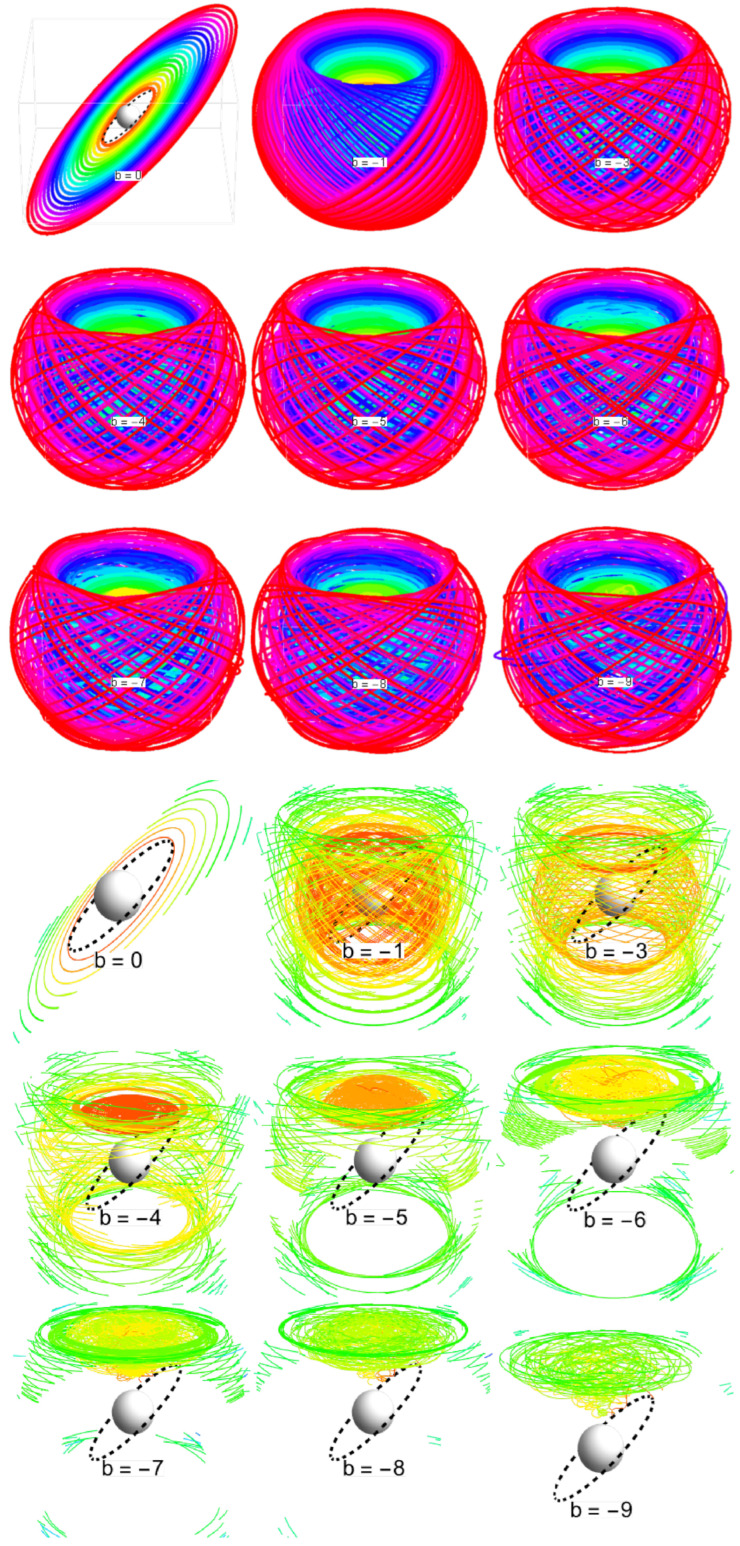
Keplerian disk inclined by θ=π/4 with small magnitude of negative parameter *b*. Upper panel: global view. Lower panel: zoom around the central object.

**Figure A6:**
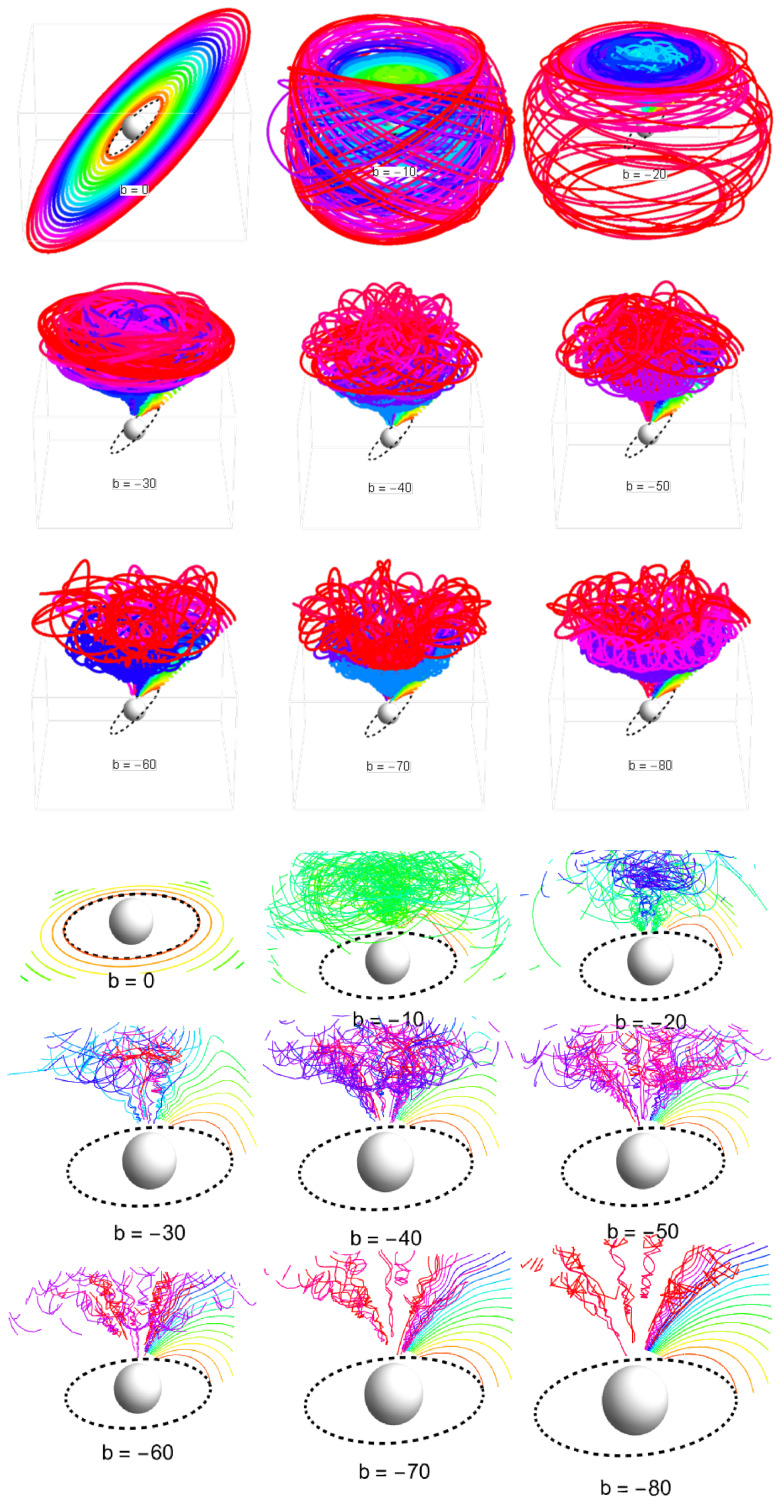
Keplerian disk inclined by θ=π/4 with large magnitude of negative parameter *b*. Upper panel: global view. Lower panel: zoom around the central object.

Highly inclined disks are ideal to illustrate the role of the violation of the axial symmetry of the magnetic field by the position of a Keplerian disk, and they enable us to demonstrate the motion in belts connected to the potential wells around stable off-equatorial circular orbits. The results are given in Figure A5 (Figure A6) for small (large) magnitudes of the magnetic parameter *b*; see Appendix A.

For highly inclined Keplerian disks, the behavior of the disk strongly depends on the magnitude of the parameter *b*. For the smallest magnetic parameter under consideration, b=−1, the ionized disk demonstrates a shift to a structure resembling a sphere created by cocentric shells, indicating motion in a gravitational regime. These shells are cut at the top of the sphere orthogonally to the magnetic field axis. These cuts represent the range of epicyclic oscillatory motion of particles starting at a radius $r>r_{ms}$. The shells arise across the whole Keplerian disk.

With increasing magnitudes of the magnetic parameter, starting at b=−4 and reaching up to b=−10, the inner part of the disk enters a chaotic regime of motion. The extent of this region increases with increasing *b*, and the motion demonstrates an increasing tendency to be concentrated in belts connected to the potential wells of the off-equatorial circular orbits. The regions of chaotic motion are enveloped by external shells corresponding to the gravitational regime of motion.

For large negative values of the magnetic parameter, at b<−10, all three regimes of motion emerge. At the innermost part of the Keplerian disk, the ionized particles are repelled at large distances along regular orbits. At more distant regions, the chaotic regime of motion is observed, with a strong tendency for concentration in belts around the off-equatorial circular orbits. Finally, a region of shells corresponding to the gravitational regime of motion is created, starting at sufficiently large distances. It must be noted that the region of chaotic motion is concentrated in “cone-like” structures for b<−40. In the inner regions of these “cones”, a “sub-cone” structure can be observed.

### 5.5. Lorentz Attraction

The Lorentz attraction in the equatorial plane (with parameter b>0) supports the action of gravitational forces; outside the equatorial plane, the behavior of the magnetic field is more complex. In our discussion, we follow the approach introduced for the case of Lorentz repulsion. However, in the case of magnetic attraction, the instability of the charge particle motion after disk ionization is related to the radial direction, in direct opposition to the repulsive magnetic force, implying vertical instability.

#### 5.5.1. Equatorial Disks

**Figure A7:**
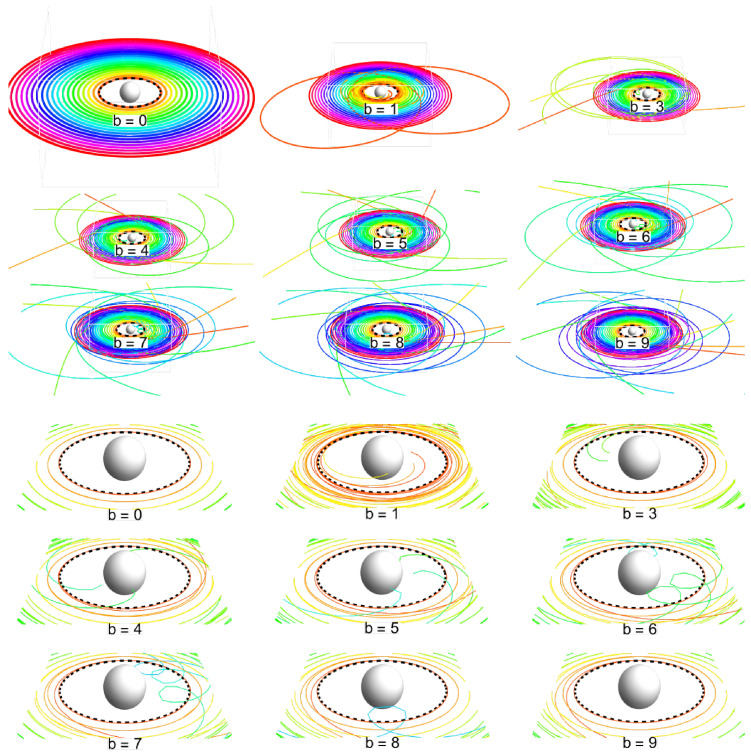
Keplerian disk on equatorial plane with small positive values of parameter *b*. Upper panel: global view. Lower panel: zoom around the central object.

**Figure A8:**
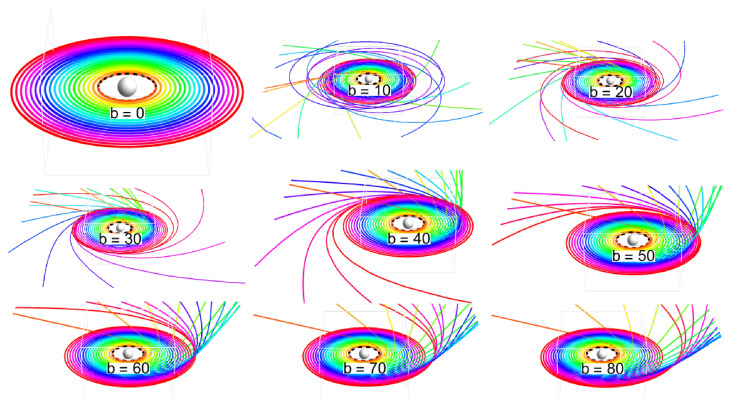

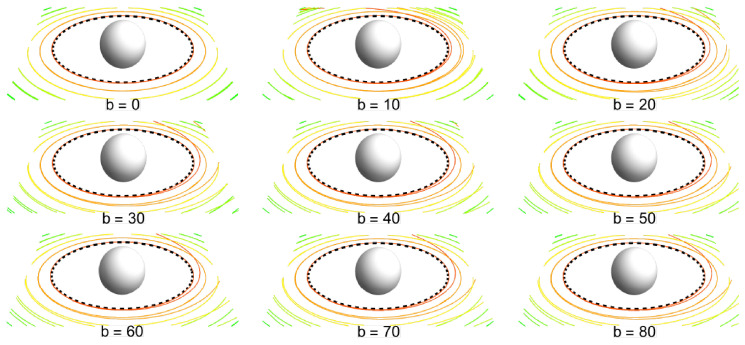
Keplerian disk on the equatorial plane with large positive values of parameter *b*. Upper panel: global view. Lower panel: zoom around the central object.

The resulting fate of the Keplerian disk is represented in Figure A7 for small magnitudes of the magnetic parameter *b* and in Figure A8 for large magnitudes of this parameter; see Appendix B. This allows a clear illustration of the emergence of the magnetic and chaotic regimes depending on the magnetic parameter *b*.

Considering small values of *b*, in the interval of (1<b<10), corresponding to the chaotic regime of motion, we observe a tendency for orbits near the geodesic $r_{ms}$ to fall onto the compact object surface along a direct trajectory, corresponding to the gravitational regime. This is observed for b<5, while, for b>5, there remains a tendency to fall onto the surface, but the trajectories exhibit loops, demonstrating the role of the magnetic field. At intermediate distances from $r_{ms}$, the orbits of charged particles of the ionized disk have a quasi-elliptic nature and demonstrate increasing chaos with increasing *b*. At large distances from $r_{ms}$, the particle motion demonstrates small epicyclic oscillations, corresponding to the gravitational regime.

For large values of b>10, particles from the inner region of the Keplerian disk exist in the magnetic regime of motion, moving along regular orbits across large distances. Meanwhile, in the intermediate region, chaotic motion is observed; at large distances, the standard gravitational regime always occurs. No fall onto the central object is observed for large values of *b*.

#### 5.5.2. Slightly Inclined Disks

**Figure A9:**
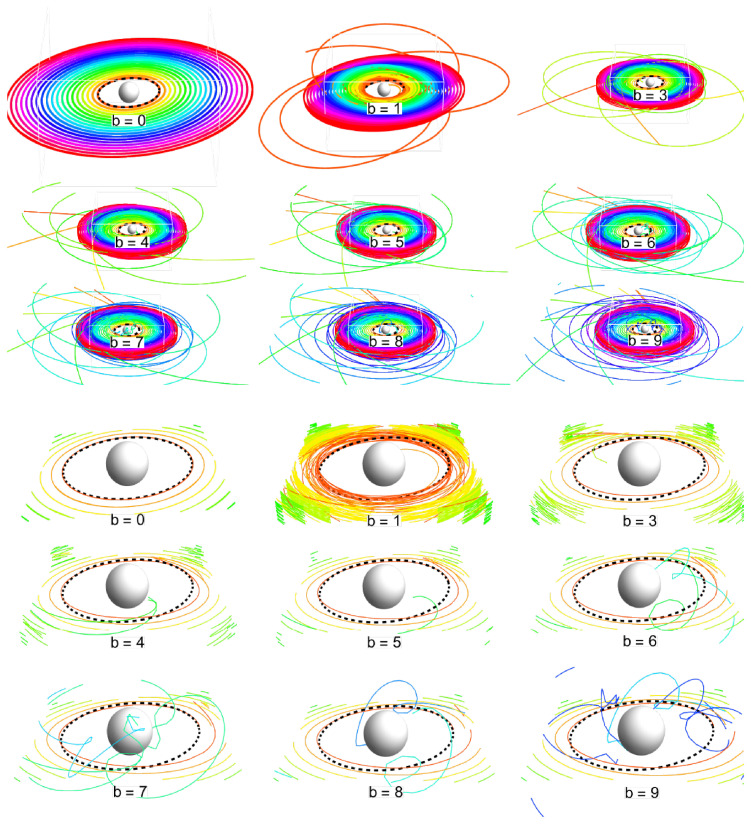
Keplerian disk slightly inclined to the equatorial plane (θ=1.5) with small positive values of parameter *b*. Upper panel: global view. Lower panel: zoom around the central object.

**Figure A10:**
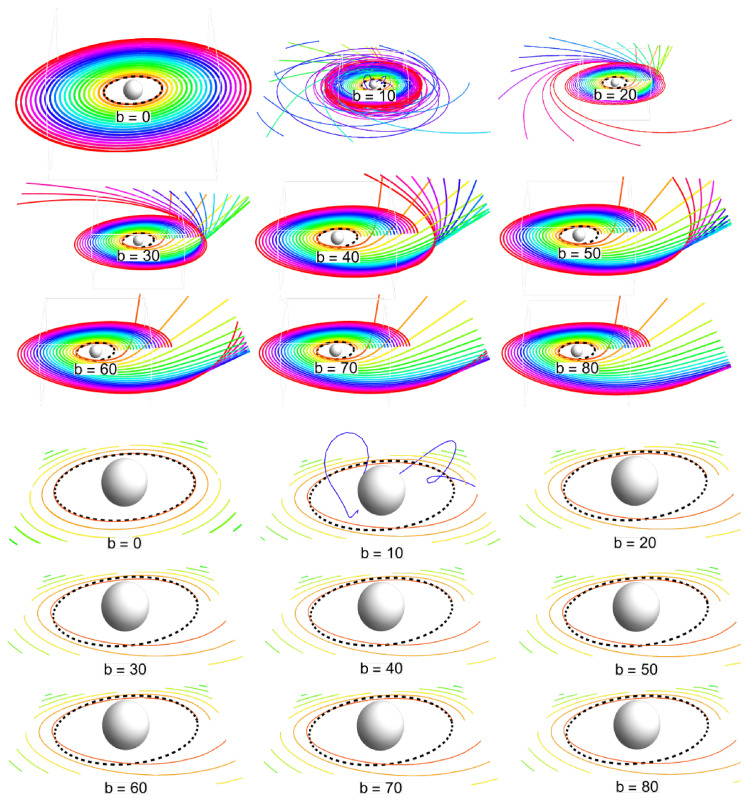
Keplerian disk slightly inclined to the equatorial plane (θ=1.5) with large positive values of parameter *b*. Upper panel: global view. Lower panel: zoom around the central object.

We next consider the influence of slight inclinations on the fates of ionized disks under magnetic attraction to clarify their role, as indicated in the case of exactly equatorial orbits. The resulting fate of the slightly inclined Keplerian disk is represented in Figure A9 for small magnitudes of the magnetic parameter *b* and in Figure A10 for large magnitudes of this parameter; see Appendix B. The results of the calculations are presented in Figure A9 and Figure A10 and confirm our hypothesis, mirroring the results obtained for the exactly equatorial Keplerian disk. The only difference is the more significant chaotic nature of the motion in the corresponding regime and the stronger inclination of the regular orbits, representing the magnetic regime of motion arising in the innermost parts of the Keplerian disk.

#### 5.5.3. Highly Inclined Disks

**Figure A11:**
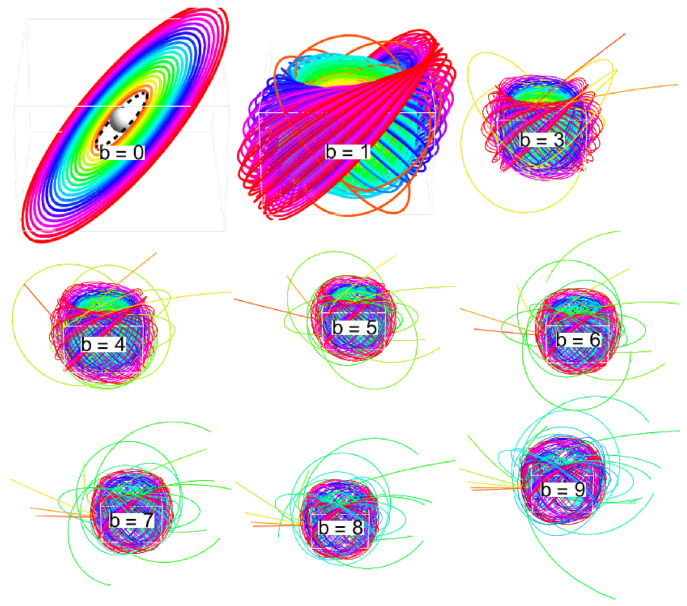

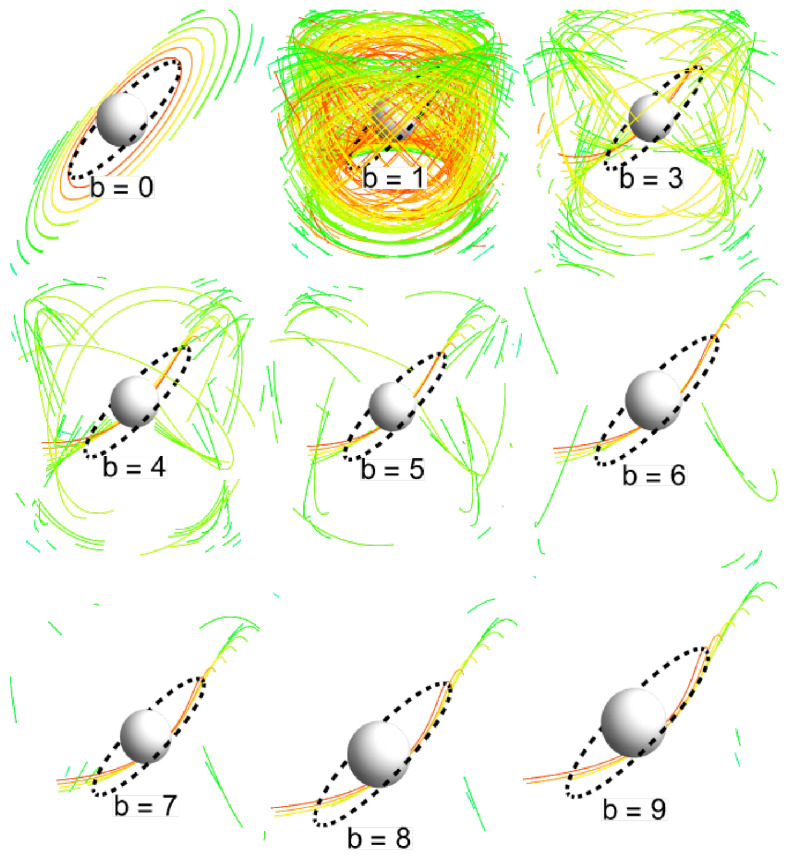
Keplerian disk inclined by θ=π/4 with small positive values of parameter *b*. Upper panel: global view. Lower panel: zoom around the central object.

**Figure A12:**
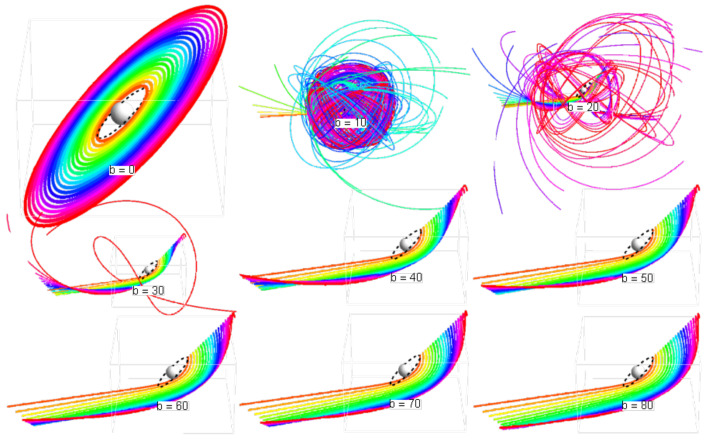

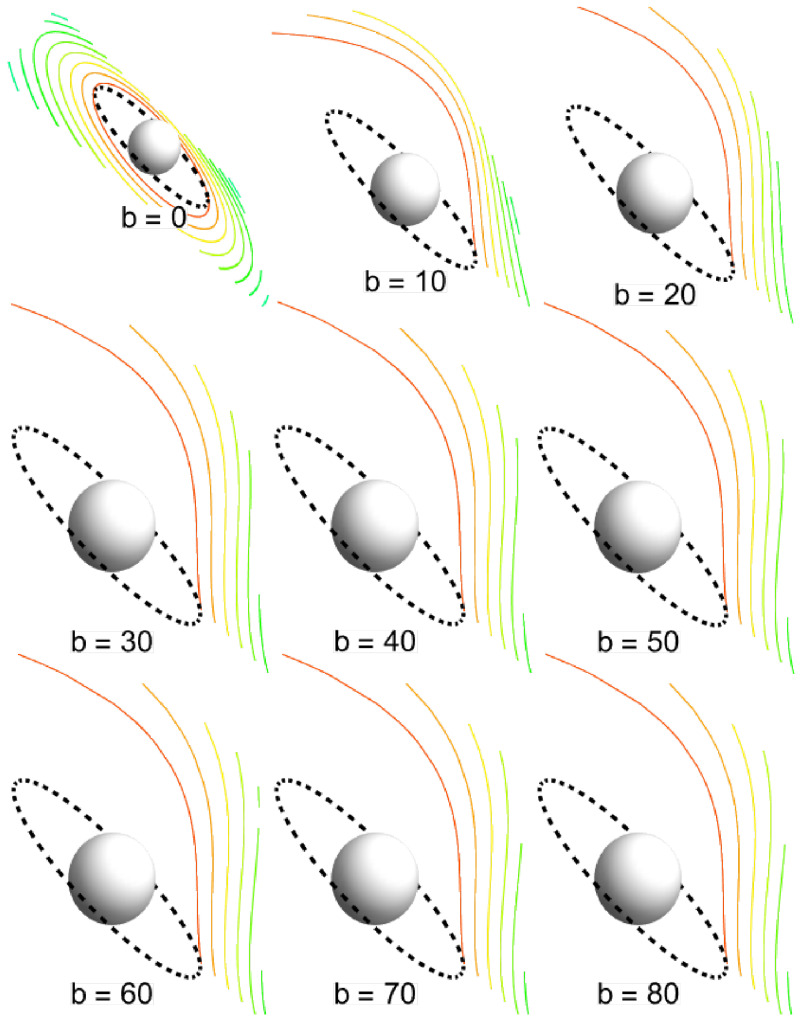
Keplerian disk inclined by θ=π/4 with large positive values of parameter *b*. Upper panel: global view. Lower panel: zoom around the central object.

We posit that highly inclined Keplerian disks can give the clearest demonstration of the fundamental differences in the behavior of charged matter influenced by magnetic attraction in comparison with those under the influence of magnetic repulsion. The fate of the highly inclined Keplerian disk is illustrated in Figure A11 for small magnitudes of the magnetic parameter *b* and in Figure A12 for large magnitudes of this parameter; see Appendix B.

As expected, we observe strong differences in the behavior of highly inclined disks under magnetic attraction in comparison with the case of magnetic repulsion. A single exception is the case of b=1, where we observe an “onion-like” structure with internal shells hidden behind the external shells. With increasing values of parameter *b* within the range of small values (1<b<10), the inner shells are successively transformed into chaotic structures, exceeding the range of regular external shells generated at larger distances in the original Keplerian disk. No tendency toward motion concentrated in belts, as in the case of magnetic repulsion, is observed.

For large values of the magnetic parameter (10<b<100), the magnetic regime of motion is crucial for the considered Keplerian disk. The inner parts of the disk are transformed into regular, highly elliptical trajectories, surpassing the region of chaotic motion generated in the intermediate region of the Keplerian disk, as well as in its external region. In fact, this kind of behavior becomes observable even within the parameter range 5<b<10, where the magnetic regime appears to be relevant in the innermost parts of the ionized Keplerian disk. In such situations, the extension of the ionized disk is governed by the innermost parts of the initial Keplerian disk.

### 5.6. Charged Equatorial Disks Under Secondary Ionization

In order to demonstrate the interplay of gravity and magnetism in extremely strong regimes in close proximity to the gravitational radius, i.e., near the photon’s circular geodesic, we consider the case of secondary ionized equatorial disks. We initially assume an equatorial disk formed by charged matter under magnetic repulsion, with a magnetic parameter allowing for the existence of stable circular orbits located under $r_{ph}=3$. For simplicity, and in order to ensure a minimal $r_{c(ms)}=2.731$, we choose the critical magnetic parameter $b_{c(ms)}=-0.691$. We assume that this is related to Fe ions with the first level of ionization (a Fe atom without one electron). We can thus consider secondary ionization, which results in an ionized state at the level n=2,3,…56. The magnetic parameter can thus be modified to the value of $b=nb_{c(ms)}$. The relations of the ionization process can be correspondingly modified, and the calculations can be realized in the same way as for equatorial Keplerian disks, with the appropriate modification of the magnetic parameter $b=nb_{c(ms)}$.

Due to the specific nature of the problem, we consider only the case of Lorentz repulsion, as the original state of the charged disk must correspond to magnetic repulsion, and ionization can only increase the level of ionization. Moreover, only charged equatorial disks can be considered as the initial state. Therefore, we focus on the region located close to the photon’s circular geodesic, as this is the region of interest, where we assume a starting position of $r>r_{ms}=6$.

**Figure 3:**
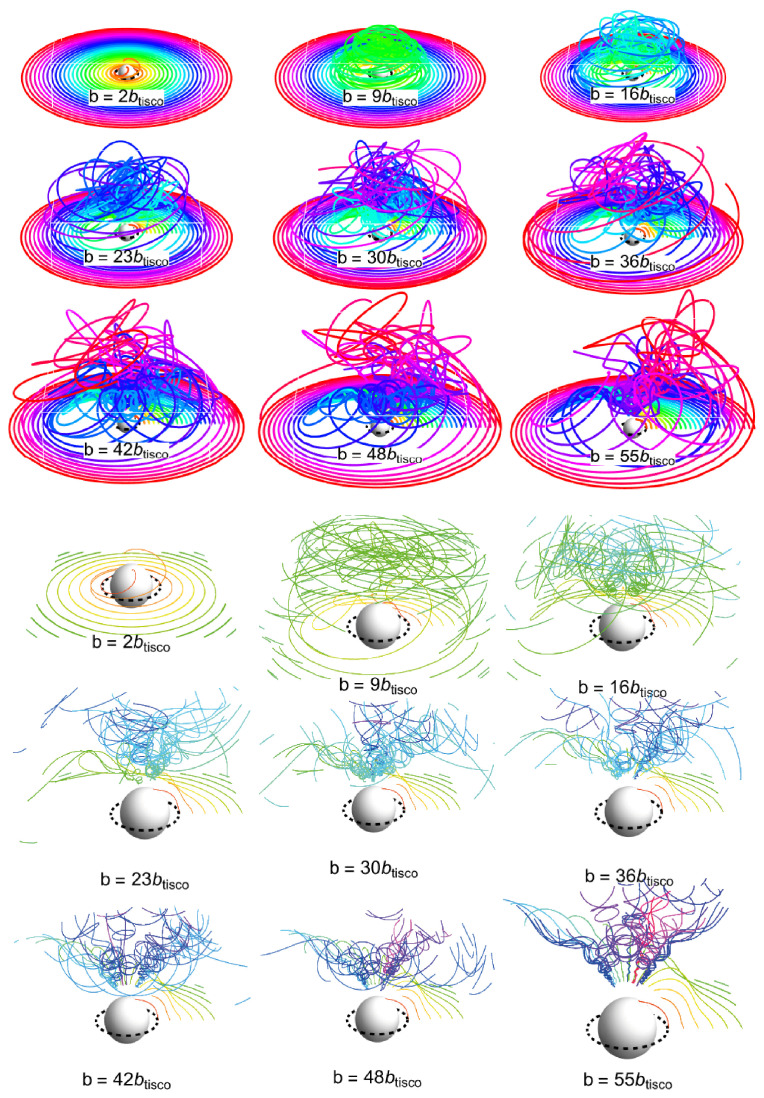
Charged equatorial disk with specific values of parameter *b*—multiples of $b_{tisco}$—given in Figure 1. The inner edge of the disk is at R=2.731. Upper panel: global view. Lower panel: zoom around the central object.

The results of the calculations are illustrated in Figure 3, covering the whole range of possible values of parameter $b\leq 56b_{tisco}$.

The results of the calculations for the secondary ionized charged disks correspond to the results obtained for the equatorial Keplerian disks, but their range is extended substantially under the marginally stable geodesic of the Schwarzschild spacetime. In the region around the photon’s circular geodesic, we demonstrate the chaotic nature of the motion, even when selecting a minimal value of $b=2b_{tisco}$.

### 5.7. Keplerian Disks Ionized in Asymptotically Uniform Magnetic Field

The electromagnetic potential of the asymptotically uniform magnetic field in the Schwarzschild spacetime field has only an axial component, which takes the form [11,12,47]

(46) $$A_{\varphi }=\frac{B}{2}g_{\varphi \varphi }=\frac{B}{2}\;r^{2}\sin^{2}\theta$$

The magnetic parameter serving as a constant of motion is determined via Equation (3). Note that, in contrast to the case of the dipole magnetic field, where the magnetic repulsion acting on corotating particles corresponds to b<0, in the uniform magnetic field, it corresponds to b>0 [4]. This difference is caused by the different characteristics of these fields.

**Figure 4:**
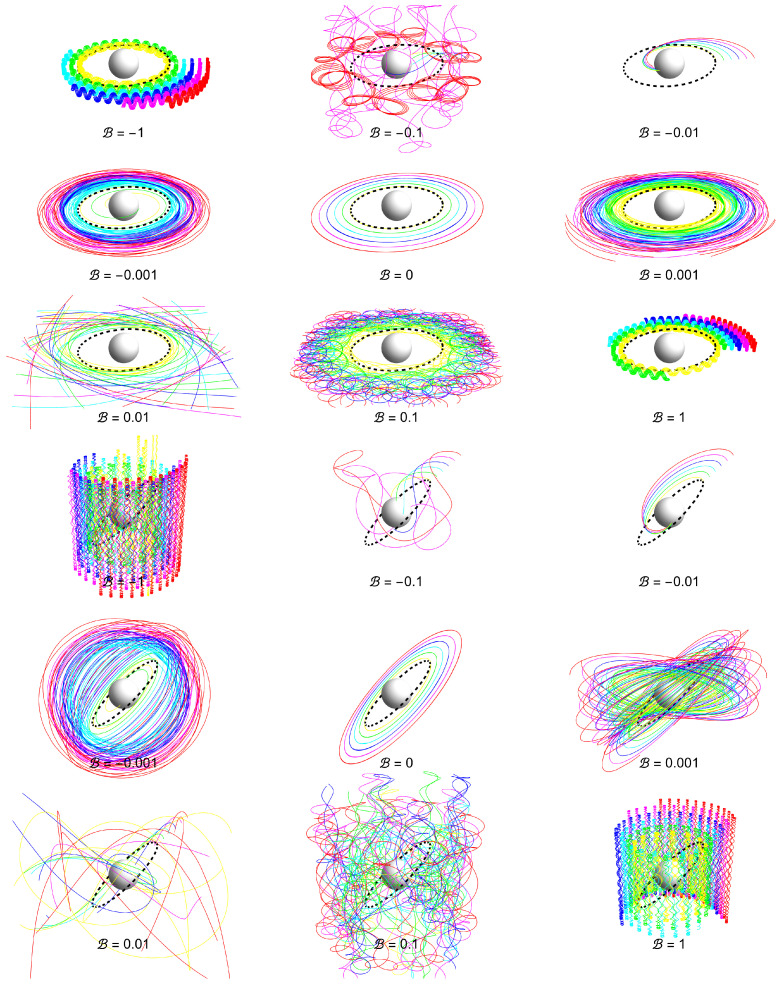
Keplerian disk in Schwarzschild background immersed in a homogeneous magnetic field with specific values of parameter *B*. Upper panel: slightly inclined (θ=1.5). Lower panel: highly inclined (θ=π/4).

The ionization of the Keplerian disks orbiting a Schwarzschild black hole immersed in an asymptotically uniform magnetic field was studied in detail in [4,12]. In Figure 4, we demonstrate the fates of the ionized disks for both Lorentz repulsion and attraction and for both slightly inclined and highly inclined Keplerian disks.

In the asymptotically uniform field, the fate of the ionized disk demonstrates signficant differences in comparison with the fate in the dipole field; this is due to the differences in the nature of the interplay of the gravitation field, vanishing at $∼1/r^{2}$, and the magnetic field. While the dipole magnetic field vanishes at $∼1/r^{3}$, implying the relevance of the gravitational regime of the charged test particle motion at large distances from the compact object, the uniform magnetic field is assumed to remain constant at large distances, implying the relevance of the magnetic regime of particle motion at large distances.

It can clearly be seen that the role of the uniform magnetic field increases with increasing distances from the central black hole, so the magnetic regime is relevant at sufficiently large distances, in contrast to the case of the dipole magnetic field, where the gravitational regime is relevant at sufficient distances. This is a crucial difference in the action of these types of magnetic fields. There are also other significant differences. We observe in Figure A7, Figure A8, Figure A9, Figure A10, Figure A11 and Figure A12 that magnetic attraction (b<0) leads to direct falling into the black hole; this phenomenon is more significant in comparison with the attractive effect of the dipole field.

We also observe differences in the nature of the chaotic regime of motion in the case of dipole and uniform magnetic fields: there is a similar reversal in supporting the chaotic nature of the motion, which is considerably stronger for the magnetic repulsion of the uniform field in comparison with the dipole field.

## 6. Discussion and Conclusions

Keplerian disks ionized via appropriate irradiation can serve as an illustrative model demonstrating the interplay of the gravitational and electromagnetic fields governing the charged test particles’ motion around black holes (or other extremely compact objects) immersed in an external magnetic field or endowed by an electromagnetic field. Such models can be useful in estimating the collective phenomena related to charged particle motion in complex astrophysical situations observed in proximity to extremely compact objects. A special case of ionized disks can be derived from satellites (comets, planets, stars) orbiting a central compact object—tidal forces in close proximity to a compact object can destroy, or partially destroy, satellites. In this way, inclined ionized disks can be formed naturally. Of course, in the detailed modeling of astrophysical phenomena in such complex situations, more advanced theories, such as kinetic theory or magnetohydrodynamics, are required, but our model could serve as a useful aid for their understanding.

Here, we illustrate the influence of the gravomagnetic field, represented by the Schwarzschild gravitational field combined with a dipole magnetic field, by considering in detail the behavior of ionized Keplerian disks. In such a combined field, three regimes of charged particle motion are relevant (gravitational, chaotic, and magnetic), governed by the intensity of the influences of gravity and magnetism. Their interplay is efficiently represented by ionized Keplerian disks. In particular, the major differences in the actions of magnetic repulsion and attraction in a given background are illustrated through the fates of ionized Keplerian disks, enabling an understanding of the physical origins of collective phenomena related to the motion of charged matter around compact objects.

There are several signatures of dipole magnetic fields in astrophysical phenomena. In particular, there is a significant difference in the behavior of ionized disks under magnetic repulsion and attraction.

In the case of magnetic repulsion (with magnetic parameter b<−0.691), the behavior of the disk is governed by instability in the vertical direction and by existence of stable off-equatorial circular orbits, allowing for motion in belts that are restricted from above by the radius $b_{\theta ISCO}\left(r\right)$, while direct falling onto the compact object is not allowed. For small magnitudes of *b*, hill-like structures with chaotic interiors are created; with increasing magnitudes of *b*, the chaotic region is extended and shifted to larger distances from the compact object. We can conclude that the extension of the region of the effect of chaotic motion in belts, related to off-equatorial circular orbits, increases with increasing |b| as $r_{tisco}\left(b\right)∼{\left(-b\right)}^{2/3}$. For increasingly large values of |b|, the observed structures are simply shifted to larger distances, retaining their characteristics—such behavior can be considered a signature of the scaling (self-similarity) symmetry that is relevant for very large values of |b|. The scaling symmetry requires more detailed study in the case of fully chaotic types of motion, which is planned for the future.

In the case of magnetic attraction (even for b>−0.691), the behavior of the disk is governed by radial instability, allowing direct falling onto the central object; there is no motion in belts, as no off-equatorial circular orbits are allowed in this case. The observed structures combine relatively regular motion in the magnetic regime (with elliptic-like orbits with large eccentricity) and gravitational regime (orbits with small eccentricity) with fully chaotic motion. The orbits related to the magnetic regime cross the region of chaotic motion.

The most important observed effect is the one represented by the fates of inclined Keplerian disks, which give a direct indication of the existence of off-equatorial circular orbits due to the creation of belts of a conical type in the region containing particles in the chaotic regime of motion—if b≤−20. This is a crucial property of the magnetic repulsive effect. Another important effect of magnetic repulsion is the existence of stable equatorial orbits and related chaotic motion in their vicinities, which could be extended to the close vicinities of extremely compact objects, namely to the region located around the photon circular geodesic—see [13]. On the other hand, in the case of inclined disks under magnetic attraction, for large values of b>20, we observe the increasing extension of the region of motion under the magnetic regime, combined with the chaotic regime, for increasing values of the parameter *b*. This effect can again be considered in the context of the scaling effect.

For weakly magnetized compact objects, an analysis of ionized Keplerian disks limited to the magnetic parameter range 1<|b|<100 is feasible. We could extend this analysis to strongly magnetized compact objects with |b|≫1, far surpassing the range applied in our numerical calculations. The behavior of the observed structures for values of |b|>30 clearly demonstrates qualitative similarity, particularly regarding the scaling self-similarity effect. This effect can thus serve as a useful guide for intuitive estimations of the behavior of ionized disks at very large magnetic parameter values.

Finally, we note that our results are relevant in the case of both black holes or their mimickers and neutron stars, as Keplerian disks have an inner radius $r_{ms}=6$, being located not only above the black hole’s horizon or its mimicker’s surface but also above the neutron star’s surface, estimated at $R_{NS}∼4$. Therefore, all discussed phenomena regarding the gravomagnetic interplay enter the considered region above all considered compact objects.

We can conclude that ionized Keplerian disks exhibit clear signatures of different types of magnetic fields, particularly dipole and asymptotically uniform ones. It is shown that these fields exhibit different characteristics at sufficiently large distances from the central object, as the regime of motion is necessarily of the gravitational type for the dipole field, while it is of the magnetic type for the uniform field. Therefore, ionized Keplerian disks can serve as a very useful model representing the nature of gravomagnetic interactions in the strong field regime.

## Figures and Tables

**Table 1:** Magnetic parameter *b* (Equation (20)) for selected magnetic field strengths for protons; black hole mass $M=10\;M_{⊙}$.

Mag. field *B* [G]	$10^{-5}$	10	$10^{4}$	$10^{8}$	$10^{12}$	$10^{15}$
$b_{\mathrm{proton}}$	$10^{-5}$	10	$10^{4}$	$10^{8}$	$10^{12}$	$10^{15}$

## Data Availability

The original contributions presented in this study are included in the article. Further inquiries can be directed to the corresponding author.
